# Tribological Properties of Ultrananocrystalline Diamond Films: Mechanochemical Transformation of Sliding Interfaces

**DOI:** 10.1038/s41598-017-18425-4

**Published:** 2018-01-10

**Authors:** Revati Rani, Kalpataru Panda, Niranjan Kumar, Kozakov Alexey Titovich, Kolesnikov Vladimir Ivanovich, Sidashov Andrey Vyacheslavovich, I-Nan Lin

**Affiliations:** 1 Materials Science Group, Indira Gandhi Centre for Atomic Research, HBNI, Kalpakkam, 603102 Tamil Nadu India; 2 Center for Nanomaterials and Chemical Reactions, Institute for Basic Science (IBS), Daejeon, 34141 Korea; 3 Southern Federal University, Rostov on Don, 344006 Russian Federation; 4 Rostov State Transport University, Rostov-on-Don, 344008 Russian Federation; 5 Department of Physics, Tamkang University, New-Taipei, 251 Taiwan Republic of China

## Abstract

Improving the tribological properties of materials in ambient and high vacuum tribo-conditions is useful for inter-atmospheric applications. Highly-hydrogenated and less-hydrogenated ultrananocrystalline diamond (UNCD) films with distinct microstructural characteristics were deposited on Ti–6Al–4 V alloy, by optimizing the plasma conditions in the chemical vapor deposition. Both the UNCD films showed less friction coefficient in ambient atmospheric tribo-contact conditions due to the passivation. This provides chemical stability to UNCD films under the tribo-mechanical stressed conditions which limits the transferlayer formation and conversion of UNCD phase into graphitization/amorphization. However, in the high vacuum tribo-conditions, highly-hydrogenated UNCD films showed low friction value which gradually increased to the higher magnitude at longer sliding cycles. The low friction coefficient was indicative of passivation provided by the hydrogen network intrinsically present in the UNCD films. It gradually desorbs and the dangling bonds are progressively activated in the contact regime, leading to a gradual increase in the friction value. In contrast, less-hydrogenated UNCD films do not exhibit low friction regime in high vacuum conditions due to the lack of internal passivation. In this case, the conversion of UNCD to amorphized carbon structure in the wear tracks and amorphous carbon (a-C) tribofilm formation on ball scars were observed.

## Introduction

Understanding the friction and wear properties of any sliding devices in ambient and high vacuum conditions are essential for their potential applications in inter-atmospheric conditions. However, controlling the friction and wear behavior of sliding devices in the vacuum condition is technically challenging. Chemically modified diamond-like carbon (DLC) and nanostructured diamond films are extremely useful for such applications. However, friction and wear properties of these films are undesirable during the run-in regime which is generally associated to the residual adsorbed contamination and high surface roughness of the film^1–4^. Moreover, after the run-in stage, friction again likely to be unstable due to the periodic chemical reactions and oxidation by the moisture and water molecules present in the ambient conditions. Interaction of dangling bonds at the sliding surfaces generates adverse adhesive/abrasive wear in high vacuum tribo-conditions. Koshigan *et al*. have shown high friction and wear in high vacuum condition of silicon oxide–doped hydrogenated amorphous carbon^5^. This was explained by the transfer of iron from pin to the flat surface due to the interfacial adhesion. However, reactive gaseous atmosphere (H_2_ and O_2_) have favored the release of adhesive junction by dissociatively reacting with the mechanically-stressed sp^2^ carbon-rich surface layers and thus favors for the low friction and high wear resistance. Adhesive wear is well understood in non-hydrogenated DLC films that show high friction and wear response in vacuum tribology conditions^6^. However, such films tribologically perform well in the ambient conditions due to the effective passivation of dangling bonds at the sliding interfaces. In contrast, DLC films with the presence of intrinsic high amount of hydrogen atoms/molecules show improved tribological performance in high vacuum conditions^7,8^. In hydrogenated DLC, hydrogen atoms act to saturate the dangling bonds of carbon atoms at the contact interfaces in the vacuum tribology conditions. Tribo-test environment may cause the conversion of the carbonaceous phases and modification of chemical nature of these tribofilms which largely affect the tribological properties^9^. In this condition, improving the friction and wear properties becomes complicated due to the formation of energetically unstable sliding interfaces. The interfacial properties change periodically due to the change in roughness, deformation process, chemical reactions, oxidation and tribofilm formation^10–12^. Moreover, the conversion of tetrahedral DLC and crystalline diamond phase into amorphous carbon (a-C) and graphitic tribolayer is another important mechanism for the enhancement in friction and wear resistance properties of DLC and crystalline diamond films in humid atmosphere^13,14^. This was explained by the easy shearing mechanism of graphitic planes under tribo-stressed condition. However, at microscale tribo-contact conditions, the phase transformations or the formation of graphite-like layers were absent at the slipping interfaces of DLC/SiO_2_ pairs during the nanofretting. Here, the low friction in air was mainly attributed to high hardness and relatively lower hydrophobic nature of the DLC coating as described by Qian *et al*.^15^.

So far, tribology mechanisms such as the passivation of dangling bonds and graphitization are proposed in DLC and nanocrystalline diamond (NCD) films as mentioned above^5–9,13,14^. Additionally, the tribological properties of hydrogenated and non-hydrogenated DLC films were also well explained in earlier works. Bouchet *et al*. have investigated the tribo-induced hybridization change (sp^3^ to sp^2^) in NCD films under mild tribological contact conditions^16^. It was shown that the sp^2^ tribofilms were embedded in the diamond ultranano particles. The atomistic sliding simulation of two ultra-nanocrystalline diamond (UNCD) films showed the formation of sp^2^ rich carbon layer in vacuum condition. Recently, the ultralow friction and high wear resistance of UNCD films deposited on silicon substrate were investigated in the ambient and moist conditions^17–22^. Kumar *et al*. showed significant decrease in friction value of UNCD films with increase in humidity level^19^. This was explained by extensive passivation of carbon dangling bonds by the water vapor phase present in ambient atmosphere. Moreover, NCD films deposited on Ti-alloys showed high friction in ultra-high vacuum condition^16^. In both cases, the passivation and graphitization mechanisms were established to explain the improved tribological properties of NCD and UNCD films^16,21,23^. However, the friction and wear mechanism of UNCD films with the change in amount of intrinsic hydrogen concentration has not been studied yet in ambient and high vacuum conditions. These films are suitable for various applications including machine tools and rotating shaft seals which may operate in vacuum condition for space application^24^. The UNCD films exhibit unique physical and chemical properties due to the surface inertness, ultranano diamond grains and hydrogenated sp^2^ nanographite-like domains in the grain boundaries^17,18^. Such a unique properties are absent in nanocrystalline and microcrystalline diamond films and thus their tribological properties and operating mechanism differ from UNCD counterpart. Grain boundary features of UNCD films are unique for the improvement in various properties, including mechanical and tribological. However, friction and wear properties of UNCD films in run-in regime was adverse^17,20^. In DLC films, the removal of surface oxide layer was responsible for the observed run-in behavior^2^. The oxide contamination enhanced the defects in DLC films, resulting in the increase of surface energy. However, this regime is prolonged to longer sliding cycles in UNCD films, which might be related to the formation of mechanically stable adventitious residual layer^17^. In addition, stable and rigid asperities on the film surface might be another important reason for high friction in the beginning of the sliding cycles. This aspect was studied in DLC films^4^. The investigation of chemical properties of tribolayer and transferfilm formation in UNCD films under the ambient and high vacuum conditions will be useful to understand the friction and wear mechanism. The prominent role of graphitic phase contribution and passivation mechanism on the friction and wear behavior can be well understood together by the variation of the graphitic content in the UNCD films and performing tribology tests in two distinct conditions i.e.: (a) ambient and (b) high vacuum.

This paper reports the origin of friction and wear of two chemically different UNCD films with distinct microstructure in (a) ambient and (b) high vacuum (3 × 10^−6^ mbar) tribology conditions. The amount of hydrogen concentration in the UNCD films was manipulated in order to investigate the role of intrinsic hydrogen in UNCD films, affecting tribological properties. The amount of graphite and a-C phases were less in highly-hydrogenated UNCD films. However, the high contribution of these phases was present in less-hydrogenated UNCD films. Moreover, the grain boundary phase compositions in these films were tailored without much modifying the ultrananocrystallite diamond phase fractions. This modification was purposefully implemented in UNCD films to understand the synergistic role of sp^2^ phase fraction and hydrogen content on friction and wear behavior in two different tribology conditions. Tribochemical analysis of sliding contacts was performed in order to reveal the friction and wear mechanism.

## Results and Discussion

### Topographical and chemical characterization of films

**Figure 1:**
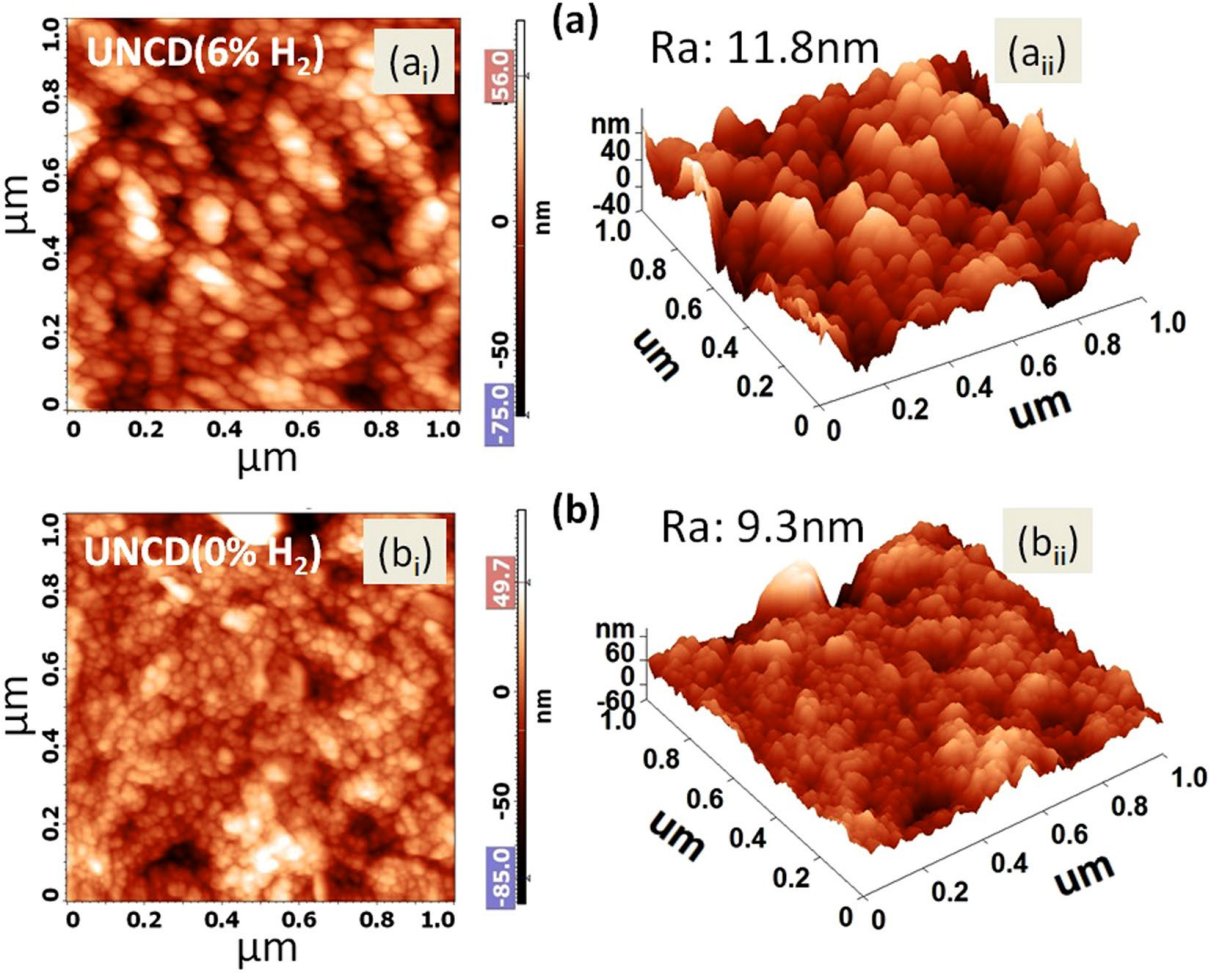
Surface topography (a_i_ and b_i_) and roughness profile (a_ii_ and b_ii_) of (**a**) UNCD(6%H_2_) (a_i_ and a_ii_) and (**b**) UNCD(0%H_2_) (b_i_ and b_ii_) films, respectively.

**Figure 2:**
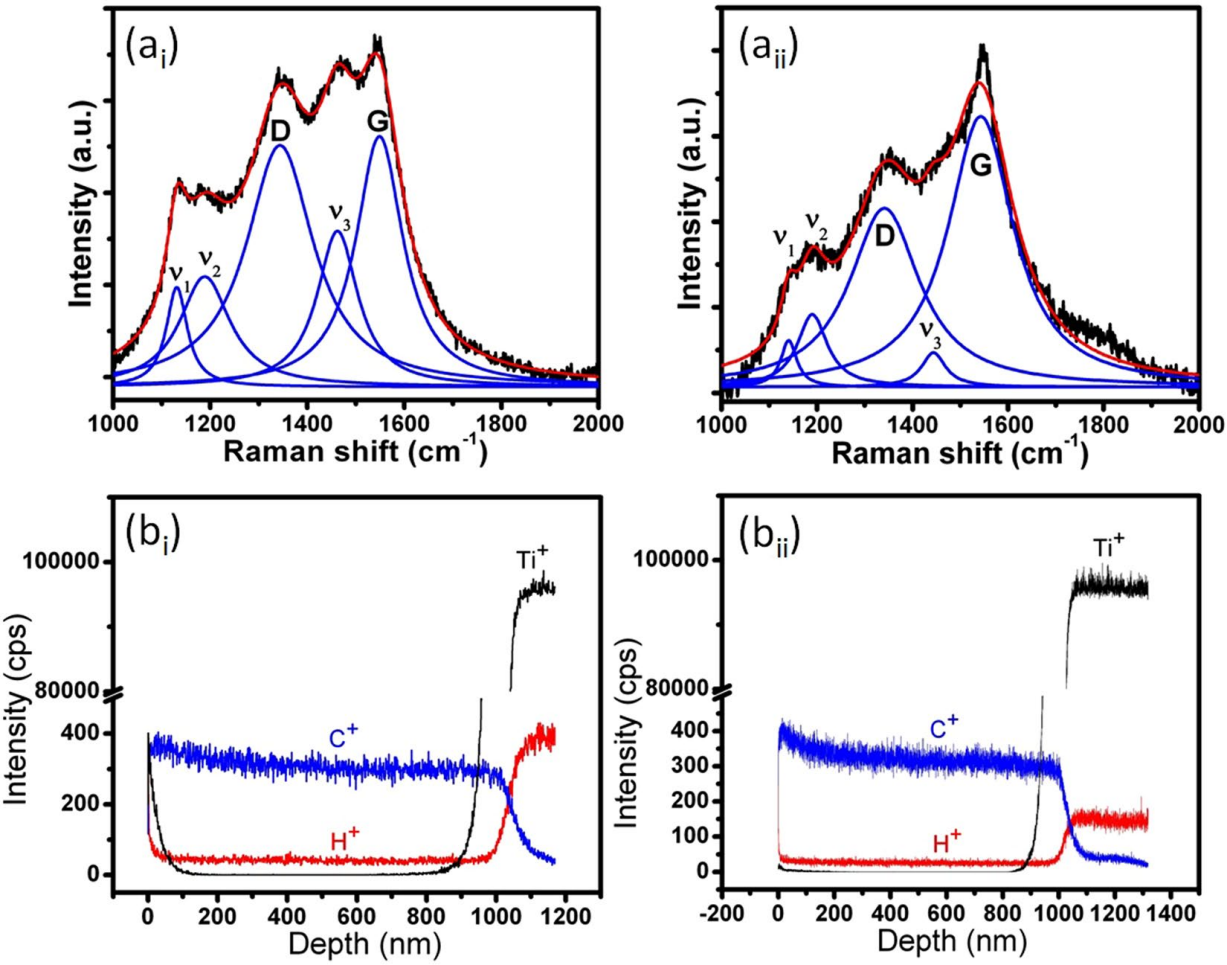
Raman spectra (a_i_,a_ii_) and SIMS depth profile (b_i_,b_ii_) of UNCD(6%H_2_) (a_i_,b_i_) and UNCD(0%H_2_) (a_ii_,b_ii_) films, respectively.

Surface topography and roughness profile of highly hydrogenated UNCD(6%H_2_) and less hydrogenated UNCD(0%H_2_) films are shown in Fig. 1a,b. Both films consist of densely packed homogeneously distributed UNCD grains. The roughness is almost similar in both the films and the grain size is smaller in UNCD(0%H_2_) films. Raman analysis is presented in Fig. 2a_i_,a_ii_ for UNCD(6%H_2_) and UNCD(0%H_2_) films, respectively. In both films, the broad Raman band can be deconvoluted into five well resolved individual bands. The diamond signature, Γ_2g_ band originating at 1332 cm^–1^, is not clearly seen in these UNCD films due to quenching of diamond band and broadening of nondiamond D band. This is the characteristics of these materials, which contain diamond grains of ultra-small size.

Peak assigned to *v*_1_, *v*_2_ and *v*_3_ are *trans-polyacetylene (TPA)* phases originating from the grain boundary of UNCD films^4,25–27^. These peaks, considered as the finger print for UNCD films^25^, and are more prominent in UNCD(6%H_2_) films. This indicates the presence of extensive hydrogenated network in the grain boundaries. Disordered carbon and graphitic domains described by the D and G bands, respectively, are known to be the major phases occupying the grain boundary regions^18,25^. This indicates the presence of amorphous and graphite phases occupying the grain boundaries^24,28^. The predominant intensity of a-C and sp^2^ phases is due to the higher cross-section of the scattered light at the visible wavelength of an exciting laser^27^. However, low scattering cross-section of the sp^3^-bonded carbon in the visible wavelength justifies the weak signal of these peaks^24^. Depth profile analysis of C, O and Ti elements are presented in Fig. 2b_i_,b_ii_ for UNCD(6%H_2_) and UNCD(0%H_2_) films, respectively. The film thickness is approximately 1 μm in both these films. The SIMS analysis clearly showed the presence of higher hydrogen concentration in UNCD(6%H_2_) films (Fig. 2b_i_) than the UNCD(0%H_2_) films (Fig. 2b_ii_). The ratio of H/C is 1:7.5 and 1:13.2 in UNCD(6%H_2_) and UNCD(0%H_2_) films, respectively. The addition of more hydrogen in the plasma medium is the reason of increased hydrogen concentration in UNCD(6%H_2_) films. The gradual change of C^+^, H^+^ and Ti^+^ at the film-substrate interfaces indicate the formation of the reactive layer. H^+^ concentration in the substrates was seen to be higher and this might be related to the residual hydrogen present on the Ti-alloy. The higher concentration of H^+^ in UNCD(6%H_2_) films compared to UNCD(0%H_2_) films, is better illustrated in the 2D and 3D elemental profiles, as given in Supplementary Information (Fig. 1SI).

### Microstructure of UNCD(6%H_2_) and UNCD(0%H_2_) films

**Figure 3:**
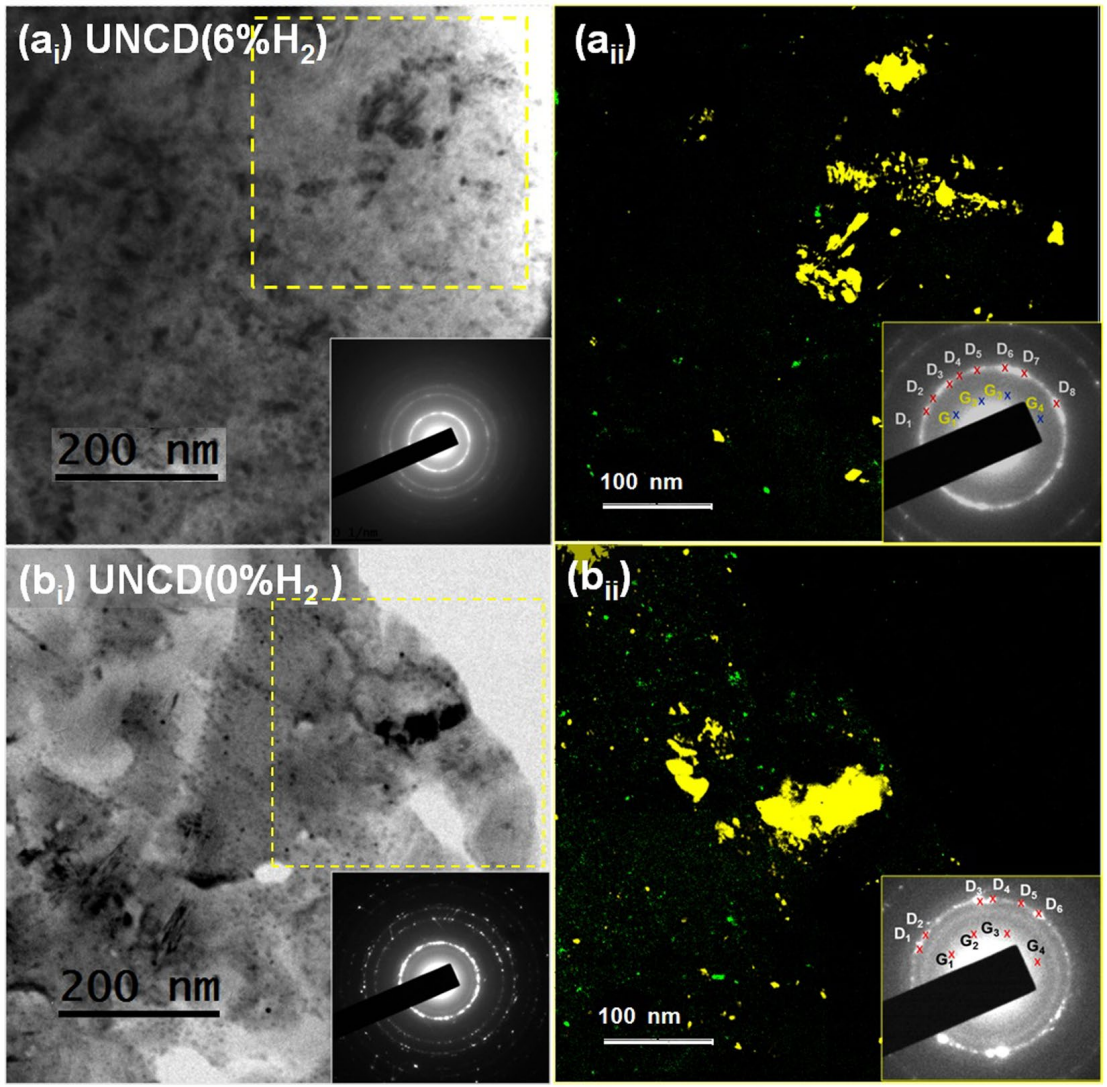
(a_i_,b_i_) Bright field (BF) TEM micrograph of (a_i_) UNCD(6%H_2_) and (b_i_) UNCD(0%H_2_); (a_ii_,b_ii_) composed dark field (c-DF) micrograph of (a_ii_) UNCD(6%H_2_) and (b_ii_) UNCD(0%H_2_) films with SAED shown as insets.

Detailed microstructure of both these films was investigated using TEM analysis. Figure 3a_i_ shows the typical bright field (BF) TEM micrograph of UNCD(6%H_2_) films with the corresponding selected area electron diffraction (SAED) patterns shown in inset. The BF(TEM) micrograph contains some darken regions, which are diamond aggregates, distributed among the matrix of ultra-small diamond grains. The SAED contains (111), (220) and (311) diffraction rings, confirming that the UNCD(6%H_2_) films are predominately diamond of Fd3m symmetry. Moreover, the SAED contains a diffuse ring in the center which implies the existence of some sp^2^-bonded carbon (a-C or graphitic phase) in these films. The distribution of the large diamond aggregates, ultra-small diamond grains and sp^2^ bonded clusters is best illustrated by the composed dark field (c-DF) micrograph shown in Fig. 3a_ii_, corresponding to the dashed square region in the BF(TEM) micrograph (Fig. 3a_i_). The c-DF micrograph is the superposition of dark-field TEM micrographs obtained using different segments of diffraction ring (designated in an inset of Fig. 3a_ii_). The yellow-colored large and small grain regions in Fig. 3a_ii_ are diamond which correspond to the diffraction spots D_1_, D_2_, …D_8_ in inset of Fig. 3a_ii_, whereas the green-colored tiny clusters are sp^2^ bonded phase which correspond to the diffraction spots G_1_, G_2_…G_4_ in inset of Fig. 3a_ii_. The diffraction spots generate D’s image corresponding to diamond in dark field (DF), obtained from diffraction spots of (111) diffraction ring and were colored yellow. In contrast, the G’s spots produce DF images corresponding to amorphous carbon. Further, for this, the diffraction spots exhibiting central diffraction ring and were colored green. The c-DF image was obtained by superimposing all the DFs images and this is shown in Fig. 3a_ii_. The other regions which show low contrast in BF(TEM) are also diamond but are oriented away from the zone-axis and thus diffract the electrons weakly. It is worth mentioning that the c-DF image has been enlarged twice as much with respect to the BF(TEM) micrographs.

The microstructure of UNCD(0%H_2_) films is shown as TEM micrographs in Fig. 3b_i_ and b_ii_ for BF(TEM) and c-DF(TEM) micrographs, respectively. The BF(TEM) micrograph of UNCD(0%H_2_) films is very much similar with those for UNCD(6%H_2_) films, indicating that the microstructure of the two UNCD films are very alike, i.e., they both contain large diamond aggregates dispersed in the matrix of ultra-small diamond grains except that the proportion of diamond aggregates in UNCD(0%H_2_) films is slightly less abundant than those in UNCD(6%H_2_) films. Such a phenomenon is more clearly illustrated in the c-DF micrographs in Fig. 3b_ii_.

**Figure 4:**
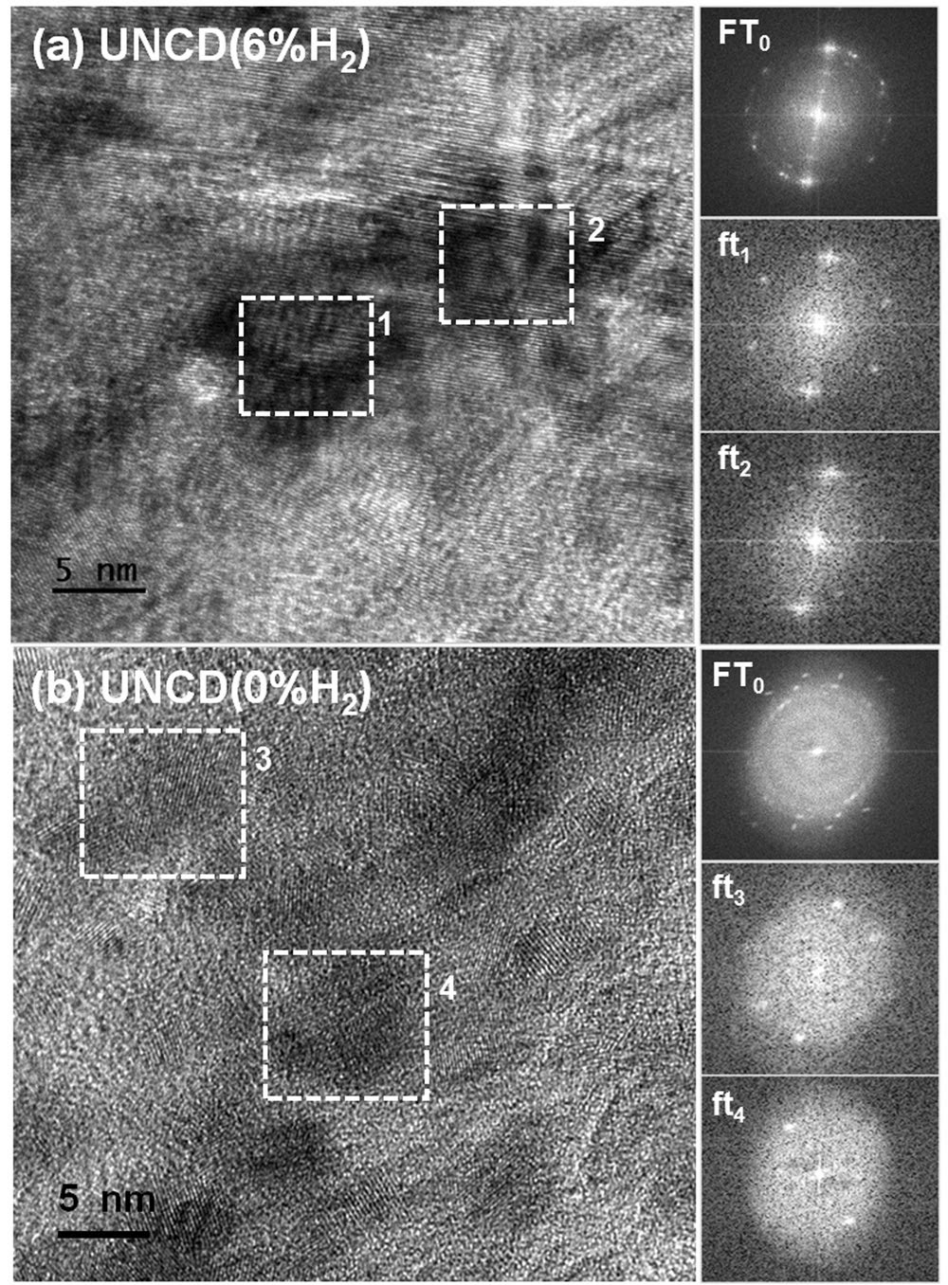
HR-TEM image of (**a**) UNCD(6%H_2_) and (**b**) UNCD(0%H_2_) films. The FT_0_ image in “a” and “b” shows the Fourier transformed diffractogram corresponding to entire structure image in “a” and “b”, whereas FT_1_ to FT_4_ are FT images corresponding to areas 1 to 4.

TEM structure image of the typical small-grain regions shown in Fig. 4, reveals the existence of ultra-small diamond grains of the size around 5 nm along with relatively wide grain boundaries for both UNCD(6%H_2_) and UNCD(0%H_2_) films. However, the microstructure of the large diamond aggregates in UNCD(6%H_2_) films are distinctly different from those in UNCD(0%H_2_) films. Figure 4a shows the structure image of large diamond aggregates in UNCD(6%H_2_) films, indicating that the parallel fringes corresponding to (111) lattice planes of grain 1 lie almost in parallel with those for grain 2 (i.e., the two grains are aligned) and the boundaries between them were completely eliminated. This is further supported by the phenomenon that the ft_1_ and ft_2_ images corresponding to regions 1 and 2 are oriented in the same direction, i.e., the adjacent diamond grains for the diamond aggregates of UNCD(6%H_2_) films have merged into single large diamond grains.

In contrast, Fig. 4b shows the structure image of UNCD(0%H_2_) films. The FT_0_ image contains diffraction spots arranged in a ring geometry, indicating that the ultra-small diamond grains are randomly oriented. The FT_0_ image contains a large central diffuse ring indicating the presence of more amorphous sp^2^ bonded carbon in UNCD(0%H_2_) films. The existence of ultra-small diamond grains is highlighted by the ft_3_ and ft_4_-images which correspond to the regions 3 and 4, respectively. It should be mentioned that other regions without lattice fringes are also diamond but they are oriented away from the zone-axis such that the lattice fringes are not visible. Notably, the diamond aggregates for UNCD(0%H_2_) films contain ultra-small diamond grains which are still isolated from each other by grain boundary phases. In contrast, the ultra-small diamond grains of diamond aggregates in UNCD(6%H_2_) films have been fused together. This occurred due to dissociation of the grain boundary phases such as a-C, sp^2^ and *TPA*.

**Figure 5:**
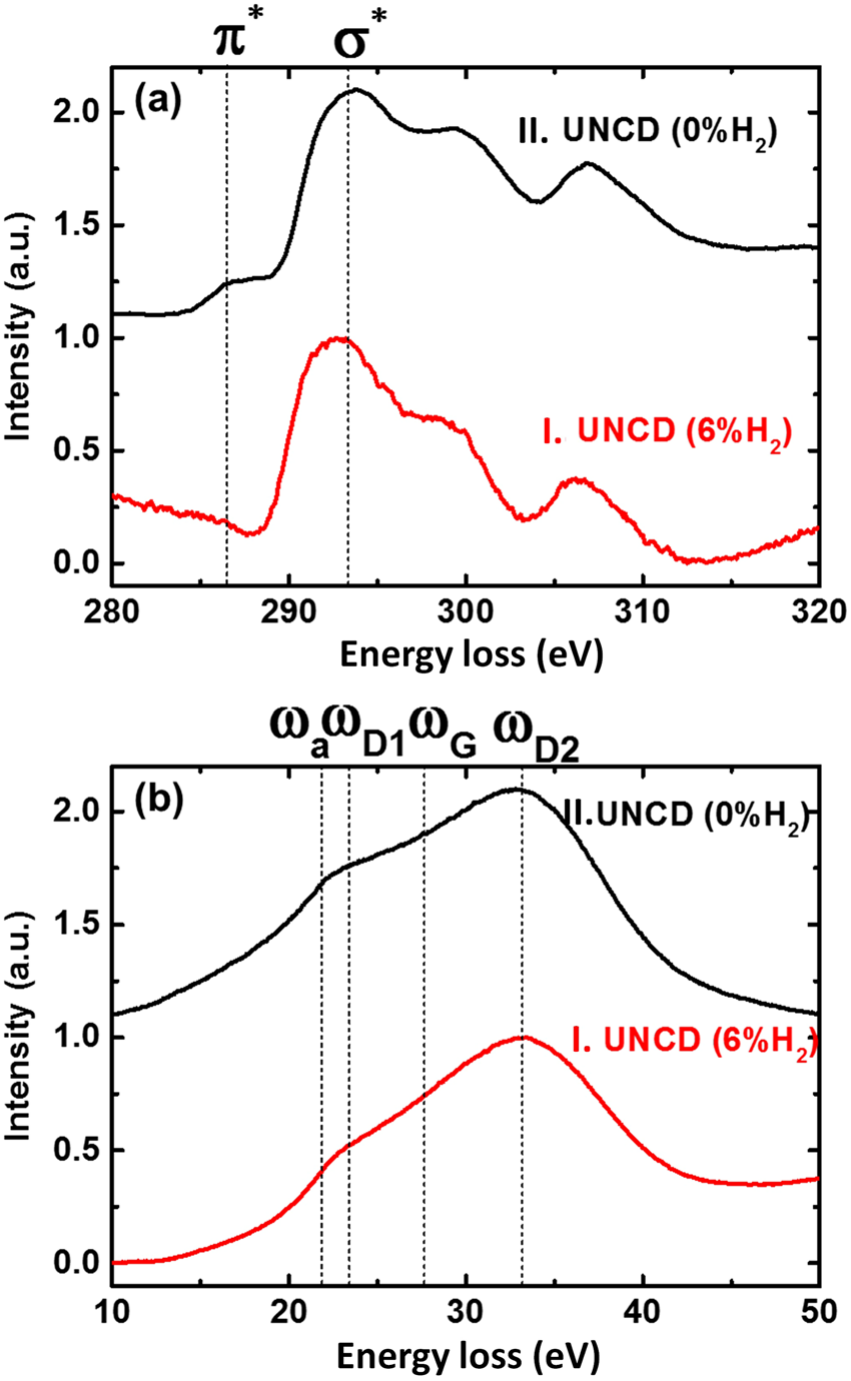
(**a**) Carbon edge core-loss EELS and (**b**) plasmon peak of (I) UNCD (6%H_2_) films and (II) UNCD (0%H_2_) films, respectively.

Electron energy loss spectroscopy (EELS) is a unique technique to characterize the local bonding nature in UNCD films as the carbon edge core-loss EELS of sp^3^-bonded carbons contain an abrupt rise near 289.5 eV (σ*-band) with a large dip near 302 eV (the secondary maximum) (Fig. 5a)^29–32^. In contrast, the sp^2^-bonded carbons contain a peak near 284.5 eV (π*-band). Furthermore, the plasmon-loss EELS can unambiguously differentiate the crystalline sp^2^-bonded carbons from amorphous ones. The graphite (crystalline sp^2^-bonded carbon) shows a peak near 27 eV (ω_G_-band), whereas the a-C phase possesses a peak near 22 eV (ω_a_-band) (Fig. 5b)^29–32^.

Moreover, the sp^3^-bonded carbon contain a peak near 33 eV (ω_D2_-band) which corresponds to the bulk plasmon of diamond materials with a shoulder at 23 eV (ω_D1_-band). This corresponds to the surface plasmon of diamond materials and the ratio of Iω_D1_/Iω_D2_ is around 1/√2. Curve I in Fig. 5a and b show the core-loss and plasmon-loss EELS spectra of UNCD(6%H_2_) films, respectively, which correspond to BF(TEM) micrographs in Fig. 3a_i_. These curves indicate that the UNCD(6%H_2_) films are predominantly diamond (sp^3^-bonded carbons) with very small content of sp^2^-bonded carbons, as the core-loss EELS contains σ*-band at 289.5 eV and secondary dip at 302 eV (curve I, Fig. 5a) with essentially no hump (π*-band) at 284.5 eV. Moreover, the plasmon-loss EELS contains ω_D1_- and ω_D2_-bands with Iω_D1_/Iω_D2_ ratio nearly 1/√2 (curve I, Fig. 5b), which implies again the content of sp^2^-bonded carbon in UNCD(6%H_2_) films is very small.

In contrast, curve II in Fig. 5a and b shows the core-loss and plasmon-loss EELS spectra of UNCD (0% H_2_) films, respectively, which correspond to BF(TEM) micrographs in Fig. 3b_i_. These curves indicate that the UNCD (0% H_2_) films are also predominantly diamond. The core-loss EELS of UNCD(0%H_2_) films contain a small hump (π*-band) at 284.5 eV, implying that these films contain more sp^2^-bonded carbon than the UNCD(6%H_2_) films (cf. curve I, Fig. 5a). Furthermore, the plasmon-loss EELS of UNCD(0%H_2_) films (curve II, Fig. 5b) contains ω_D1_- and ω_D2_-bands with the Iω_D1_/Iω_D2_ ratio slightly larger than 1/√2. The implication of such a deviation is that there exists some a-C phase besides diamond in this material and this is denoted by ω_a_-band. The observations in EELS spectroscopy for UNCD(6%H_2_) and UNCD(0%H_2_) films are in accord with those in TEM.

**Figure 6:**
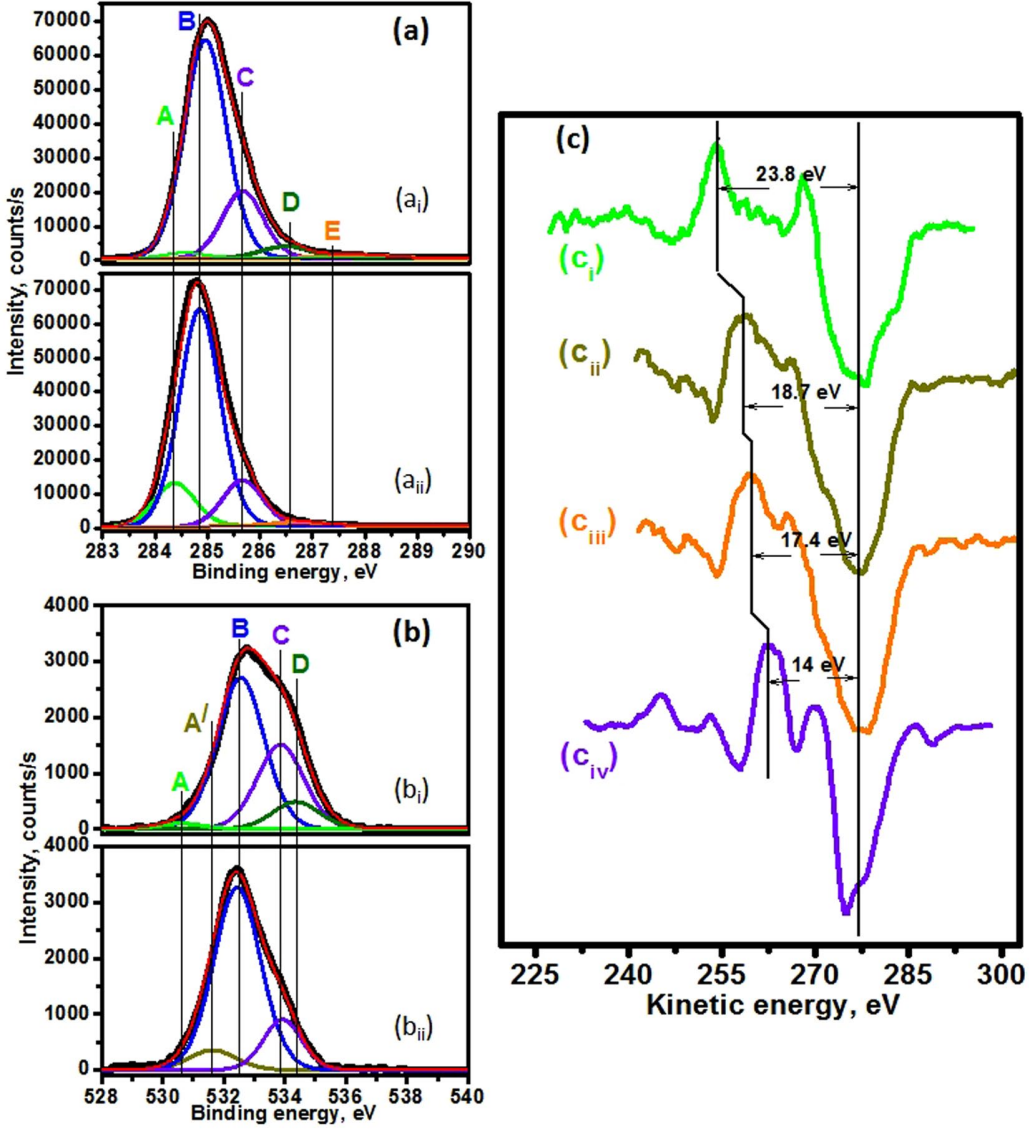
(**a**) C1s and (**b**) O1s spectra of UNCD films:(a_i_ and b_i_) are C1s and O1s of UNCD(6%H_2_); (a_ii_ and b_ii_) are C1s and O1s of UNCD(0%H_2_) films, respectively, (**c**) differential CKLL spectra of: (c_i_) highly oriented pyrolytic graphite (c_ii_) UNCD(0%H_2_) (c_iii_) UNCD(6%H_2_), and (c_iv_) polyethylene.

The Raman, TEM and EELS analyses provide valuable information of microstructure and bonding of the bulk diamond materials. However, the quantitative analysis of sp^2^ and sp^3^ phase fraction of UNCD film surface, which is more intimately related to the tribology of materials, is rather difficult. To resolve this problem, two different surface sensitive techniques (XPS and Auger) were used to differentiate the sp^2^ and sp^3^ phase fractions on the surface of two different UNCD films. XPS survey spectra (Fig. 2SI) shows the high counts of C1s peak in both these films. These counts describe 95.5 and 96.4 at% carbon in UNCD(6%H_2_) and UNCD(0%H_2_) films, respectively. Oxygen is 4.2 at% and 3.2 at% in UNCD(6%H_2_) and UNCD(0%H_2_) films, respectively. The presence of oxygen is described by the adsorption of atmospheric contamination on the film surfaces. Very small quantities of elements such as Mo, Cl, Si and S is also present on both surfaces and these elements are linked to various factors such as substrate surface preparation condition for diamond growth and contamination during sample handling. For quantitative analysis, the chemical shift of C1s and O1s peaks was investigated by the high-resolution XPS (Fig. 6a and b). The core level C1s XPS spectra shown in Fig. 6a were deconvoluted into components A and B, which represent the sp^2^ and sp^3^ network in the films, respectively^19,33^. The sp^2^ fraction in UNCD(0%H_2_) films is about 14.4% and this value is negligibly small, ~1.8%, in UNCD(6%H_2_) films. The details of the bonding fraction in at% is presented in Table 1SI. Moreover, a large amount of adsorbed oxygen functional components C (C-COO/ CH_3_COH), D (CH_2_ – O) and E (CH_2_ – O)^19^ were observed in UNCD(6%H_2_), indicating more reactive surface. Figure 6b shows that components B (OH- groups) and C (OH- groups, H_2_O) are dominating in core level photoelectron emission of O1s spectra. These components correspond to the chemical shift of hydroxyl and products of dissociated water molecules. Detail information of XPS shift of O1s spectra is given in Table 2SI. Moreover, small amount of water molecules are also adsorbed on the film surfaces. However, in XPS, depending on the surface state of diamond films, C1s energy position may vary significantly from 285 eV which makes it difficult to precisely determine the ratio of sp^3^/sp^2^ on the film surface^21^.

Auger electron spectroscopy (AES) is a complementary technique to XPS, being more sensitive to the carbon hybridization state since two valence electrons are involved in the CKLL transition. This technique is not considered sufficiently well to analyze the bulk sp^2^ phase because AES is highly surface sensitive as compared to XPS. A number of studies have shown that polyethylene (PE) CKLL Auger spectra are identical in shape to the diamond CKLL Auger spectra^34–36^. The latter technique allows to get the calibration of CKLL Auger spectra of diamond and highly oriented pyrolytic graphite (HOPG) to determine the ratio of sp^3^/sp^2^ bonds. The differentiated form of the CKLL spectrum allows measurement of the D-parameter, which gives an indication of the relative amounts of sp^2^ and sp^3^ carbon^35^. The D-parameter is 17.4 and 18.7 corresponding to 64.3 and 50.7% sp^3^ fraction in UNCD(6%H_2_) and UNCD(0%H_2_) films, respectively (Fig. 6c). For a reference purpose, the above values are compared with HOPG (100% sp^2^ bonds and 0% sp^3^ bonds) and diamond (PE) (100% sp^3^ bonds and 0% sp^2^ bonds). The UNCD (6% H_2_) films showed the high quantity of sp^3^ fraction which is well in agreement with both XPS and HRTEM. However, sp^3^ phase fractions described by AES is lower than the XPS analysis in both these films. This discrepancy arises due to the highly surface sensitive nature of AES and particular analysis indicates that surface is composed by more sp^2^ fraction compared to the subsurface. It was shown that extensive sp^2^ layer at the surface region of films was formed during the final steps of the deposition process when the microwave plasma was switched off^16^.

### Tribology results and mechanochemical behavior of the sliding interfaces

**Figure 7:**
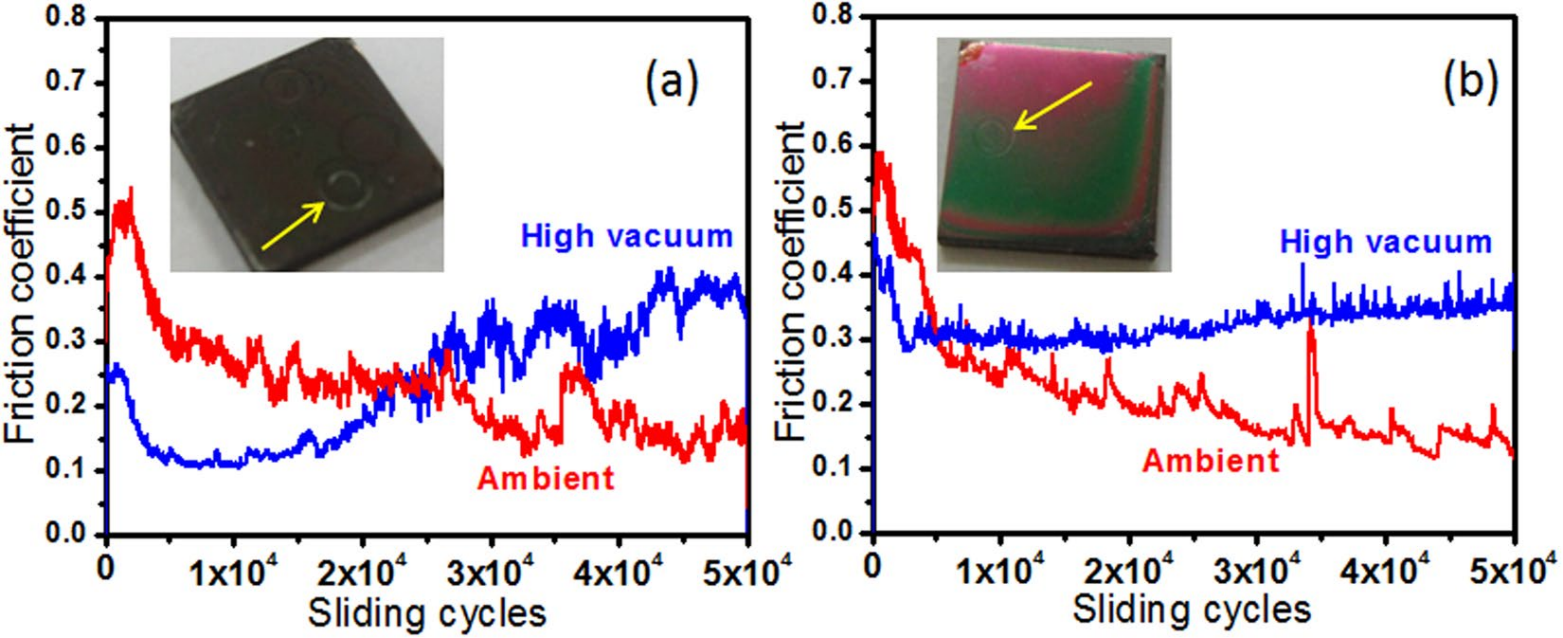
Friction coefficient (**a**) UNCD(6%H_2_) and (**b**) UNCD(0%H_2_) films in ambient and high vacuum (3 × 10^−6^ mbar) tribology conditions, respectively, with the inset showing optical images of the films exposed for tribology test and arrow marks represents wear tracks. Tribology parameters: Load: 1 N, Sliding speed: 100 rpm (linear speed: 1.04 cm/s), Ball: Al_2_O_3_ (6 mm dia).

In both the UNCD films, the value of friction coefficients were similar in ambient conditions, which gradually decreased with sliding cycles (Fig. 7). However, two distinct frictional characteristics were observed in high vacuum conditions (3 × 10^−6^ mbar). In UNCD(6%H_2_) films, the friction coefficient was lower in the beginning and gradually increased to higher value at the end of sliding cycles. Here, it shows the presence of two distinct, i.e., low and high, friction regimes. On the other hand, in UNCD(0%H_2_) films, the friction coefficient in vacuum condition was high from the beginning and does not change much even after longer sliding cycles.

**Figure 8:**
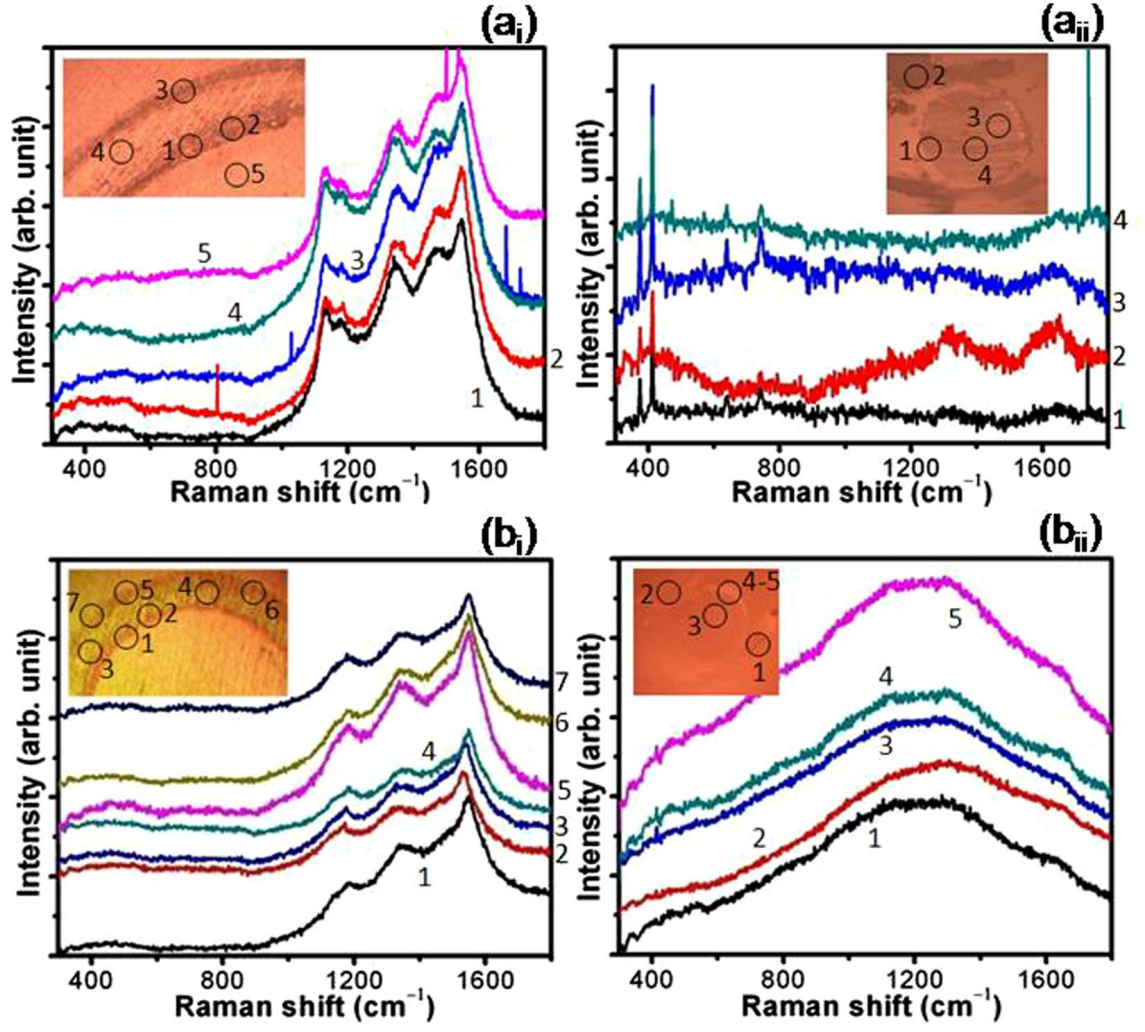
Raman spectra at various locations: (**a**_**i**_ and **b**_**i**_) wear track and (a_ii_ and b_ii_) ball scar in the sliding combination of UNCD(6%H_2_)/Al_2_O_3_ (a_i_ and a_ii_), and (b_i_ and b_ii_) UNCD(0%H_2_)/Al_2_O_3_; tribology parameters: Ambient condition, Load: 1 N, Sliding speed: 100 rpm (linear speed: 1.04 cm/s), Ball: Al_2_O_3_ (6 mm dia.), sliding cycles 5 × 10^4^.

In order to explain such a different behavior, quantitative analysis of the wear tracks and ball scars were carried out by micro-Raman spectroscopy. Raman results in Fig. 8a_i_ and b_i_ show that the chemical structure of the wear tracks is stable and does not transform into a-C/graphite phase in ambient tribology conditions regardless of the characteristics of UNCD films. However, Fig. 8a_ii_ shows a weak signal of broader peaks around 1320 cm^–1^ (D band) and 1620 cm^–1^ (G band), indicating the a-C structure^37^ at the edge of the ball scar i.e. away from the main interaction area in case of UNCD(6%H_2_) films. However, the variation in intensity of the Raman peak in this figure is related to the thickness of transferlayer. These peaks are not observed at the ball scar in the sliding combinations of UNCD(0%H_2_)/Al_2_O_3_ but shows unusual shape of the peak (Fig. 8b_ii_). This might be possible that the transfer carbon layer is quite thin in sliding combinations of UNCD(0%H_2_)/Al_2_O_3_ which give rise to a strong luminescence of alumina peak showing large background with unusual shape. Furthermore, micro-Raman spectra at various sliding cycles of 2 × 10^3^, 5 × 10^3^ and 1 × 10^4^ were also obtained as shown in Fig. 3SI. With the increase in sliding cycles, gradual formation of a-C transferlayer on the ball scar was noticed in these spectra.

**Figure 9:**
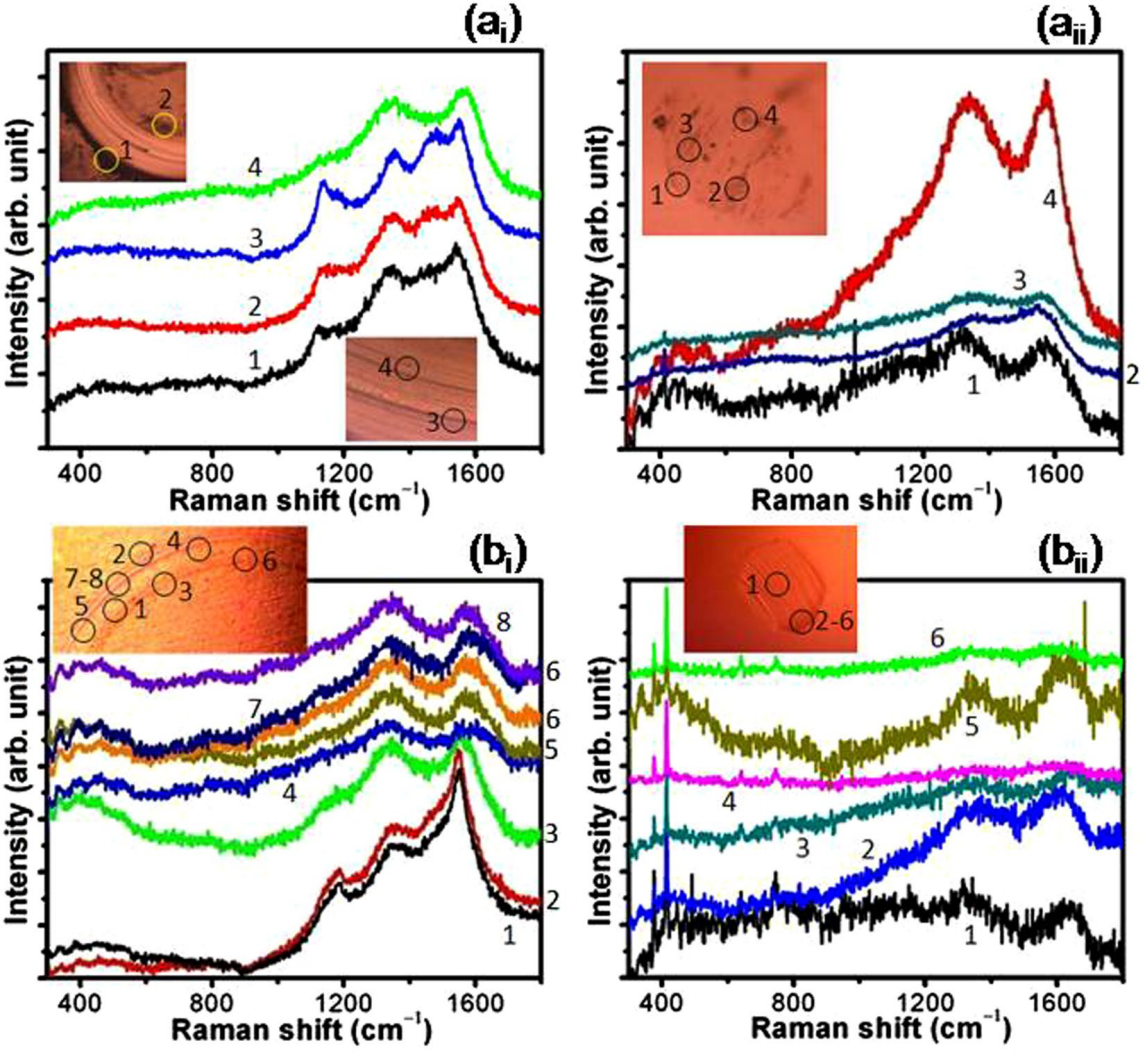
Raman spectra at various locations: of (a_i_ and b_i_) wear track and (a_ii_ and b_ii_) ball scar in the sliding combination of UNCD(6%H_2_)/Al_2_O_3_ (a_i_ and a_ii_), and (b_i_ and b_ii_) UNCD(0%H_2_)/Al_2_O_3_; tribology parameters: High vacuum (3 × 10^−6^ mbar), Load: 1 N, Sliding speed: 100 rpm (linear speed: 1.04 cm/s), Ball: Al_2_O_3_ (6 mm dia.), sliding cycles 5 × 10^4^.

Figure 9 indicates that the chemical structure of wear track in both the films was largely transformed into a-C/graphite phase in high vacuum conditions. In the wear track, this transformation was found to be prominent in UNCD(0%H_2_) films (Fig. 9b_i_) where the combination of five-peaks was transformed to a strong and broader doublet around 1345 cm^–1^ and 1570 cm^–1^ with the disappearance of *TPA* peaks. Similarly, for UNCD(6%H_2_) films, at few locations, the five peaks in Raman spectra were transformed to a broader doublet (Fig. 9a_i_). This indicates the transformation of the nanostructured diamond to a-C/graphite phase in the tribo-interaction region of UNCD films. In both ball scars, evidence of a-C transferlayer was prominently observed at high vacuum conditions and this was associated to unstable UNCD structure at contact interfaces in the absence of passivation (Figs 9a_ii_ and b_ii_). Moreover, well resolved and strong peak of a-C (Fig. 9a_ii_, curve 4) is related to the intense localized amorphization. Such transformation is locally possible due to the tribo-induced desorption of intrinsic hydrogen which enhances the interfacial resistance.

The Raman signature of the Al_2_O_3_ ball was observed in the range of 350 cm^–1^ to 800 cm^–1^ ref.^38^ and these bands are clearly seen at the ball scars. This is also well supported by spectra obtained from the virgin Al_2_O_3_ ball surfaces (Fig. 4SI). A weak feature of Al_2_O_3_ peaks was observed at the wear track, indicating the mass transfer from ball to the wear track of UNCD film during the sliding process.

**Figure 10:**
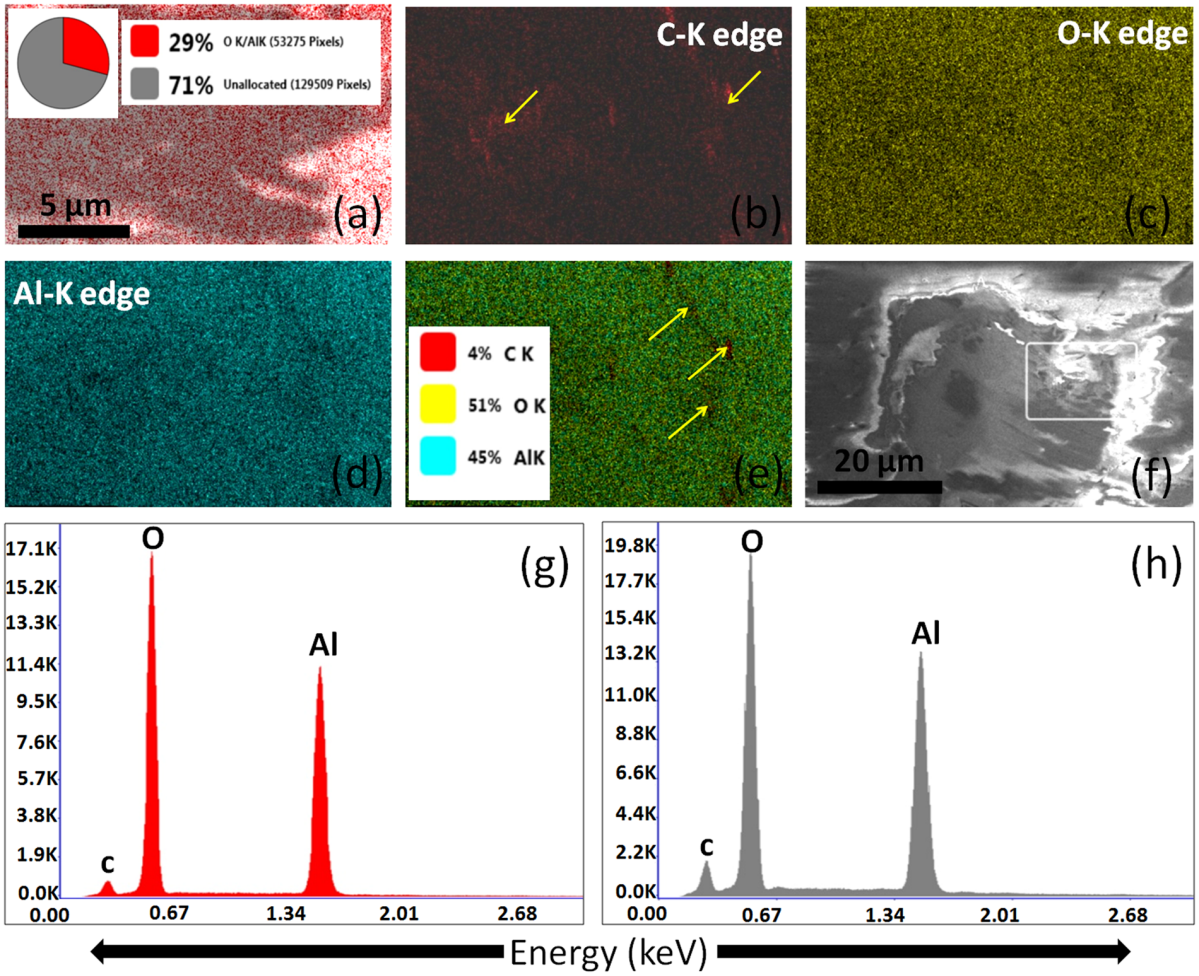
EDX elemental imaging at the ball scar of UNCD(0%H_2_) films in ambient: (**a**), (**b**) C-K edge, (**c**) O-K edge, (**d**) Al-K edge, (**e**) superimposed imaging of all elements, (**f**) backscattered image of ball scar where selected square area shows imaging region and (**g** and **h**) EDX spectra from the red and gray region located in an inset as the pie chart in figure (**a**).

**Figure 11:**
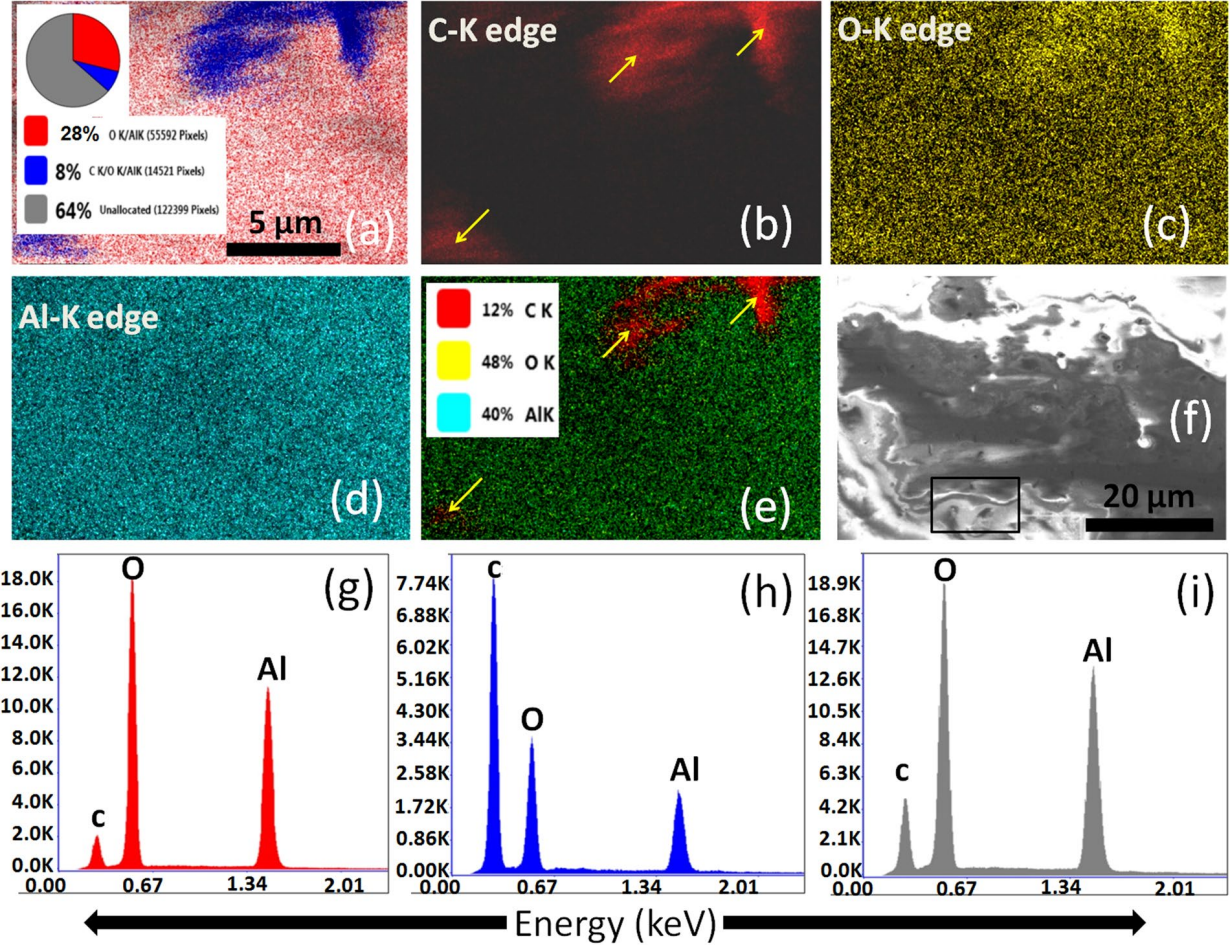
EDX elemental imaging at the ball scar of UNCD(0%H_2_) in vacuum: (**a**), (**b**) C-K edge, (**c**) O-K edge, (**d**) Al-K edge, (**e**) superimposed imag of all the elements, (**f**) backscattered image of ball scar where selected square area shows imaging region and (**g, h** and **i**) EDX spectra from red, blue and gray region, respectively, located in an inset as pie chart of figure (**a**).

Moreover, energy dispersive X-ray (EDX) mapping and XPS analysis at the deformed ball scars were also carried out. This was performed for the quantitative analysis of transferlayer formed at the Al_2_O_3_ ball scar in ambient and vacuum conditions while sliding against the UNCD (0% H_2_) films. The EDX mapping results of the ball scars in ambient and vacuum conditions is shown in Figs 10a–h and 11a–i, respectively. The analysis was carried out at the ball scar region as marked in Figs 10f and 11f. Mapping analysis showed that the concentration of carbon is higher at ball scar formed in the vacuum condition (Fig. 11). This is also well evident in the superimposed mapping image in Figs 10e and 11e. The carbon signal in both the images is clearly represented by the arrow mark (Figs 10b and 11b). Moreover, elemental spectra of carbon and other elements together in the given region are shown in Figs 10g and h and 11g,h and i. Oxygen and Al concentrations are lower while carbon concentration is high, indicating the formation of significantly carbon enriched transferlayer (Fig. 11). Quantitative EDX spectra obtained from different locations of the ball scar indicated in the pie chart in inset of Figs 10a and 11a also showed the higher intensity of carbon signal in vacuum condition.

The blue region in this chart indicates the percentage of carbon and representative EDX spectra in this region also highlights the higher intensity of carbon (Fig. 11h). Other regions have also the higher carbon quantity in this particular scar (Fig. 11g and i).

**Figure 12:**
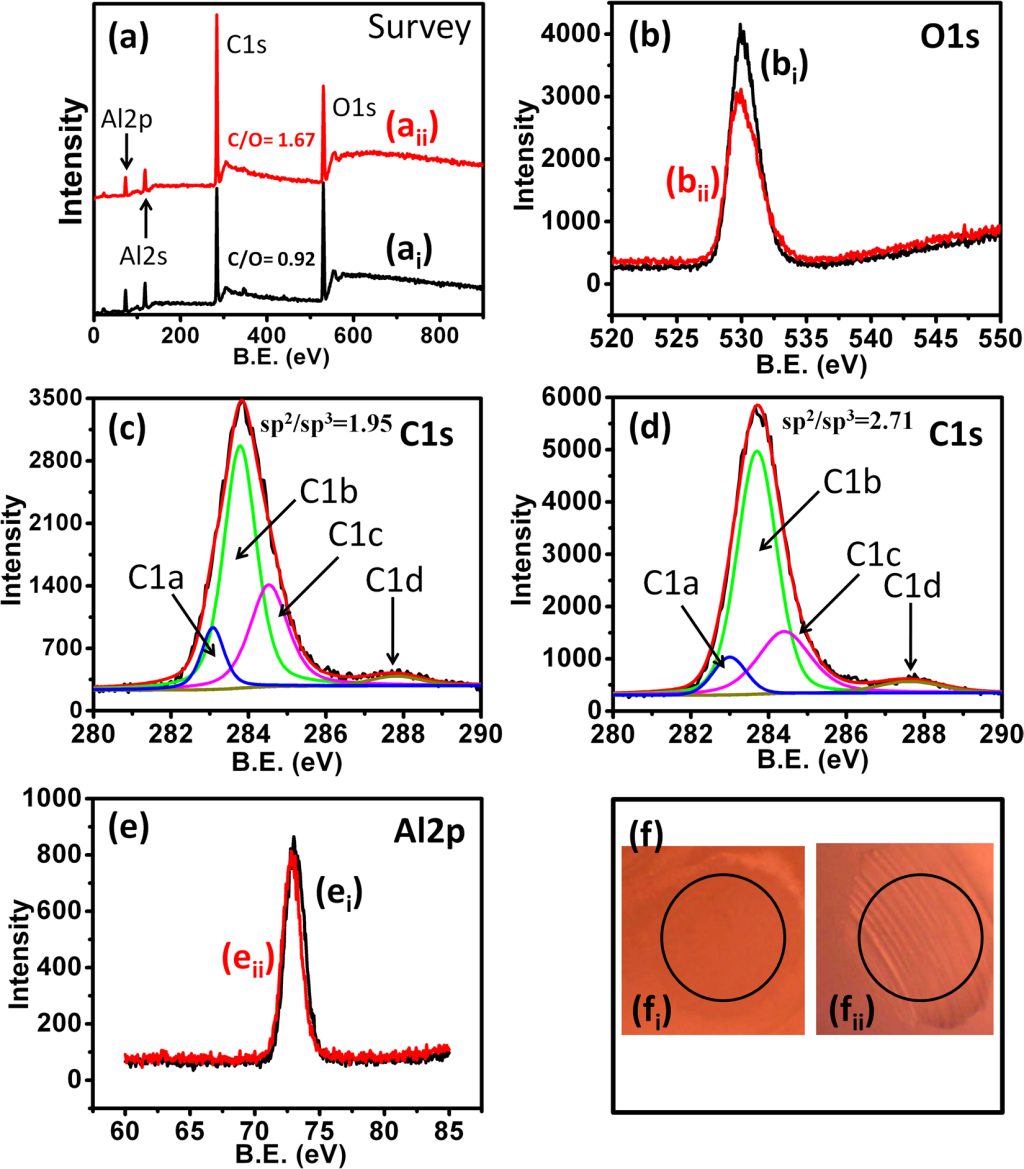
Survey XPS spectra at the Al_2_O_3_ ball scar formed against the UNCD(0%H_2_) films sliding in two different tribo-condition: ambient (a_i_) and high vacuum (a_ii_) condition, (**b** to **e**) high resolution XPS: O1s peaks in ambient (b_i_) and high vacuum (b_ii_), C1s peaks in ambient (**c**) and in high vacuum (**d**) and Al2p peaks in ambient (e_i_) and in high vacuum (e_ii_), optical micrograph of ball scars where circles show x-ray exposed region during XPS: (f_i_) in ambient and (f_ii_) in high vacuum conditions.

Quantitative XPS analysis at the deformed ball scars is shown in Fig. 12. Intentionally, the whole region of the ball scar was exposed by the X-rays in order to obtain the averaged XPS results. The X-ray exposed region is typically indicated by circle in Fig. 12f. For comparison, similar conditions and parameters were used for XPS analysis of both the ball scars. The XPS survey spectra clearly shows more intensity counts of C1s peak at the ball scar formed in vacuum condition (Fig. 12a_ii_) and supports the Raman results (Fig. 9b_ii_). In this condition, the ratio of C1s/O1s is significantly high (1.67) as compared to the scar formed in ambient atmospheric condition (0.92) (Fig. 12a). This factual evidence shows large amount of oxygen at the ball scar formed in ambient conditions. Obviously, O1s peak intensity count is decreased by 22.8% in vacuum condition as shown in high-resolution XPS spectra (Fig. 12b_ii_). Further, high-resolution C1s peak is deconvoluted into four individual peaks for detail chemical shift analysis of carbon environment (Figs 12c and d). The first one (C1a) at lower binding energy could be associated with charging effect^39^. Other three peaks C1b, C1c and C1d are related to sp^2^, sp^3^ and carboxylic groups, respectively^19,33^. In both tribology conditions, sp^2^/sp^3^ fraction at the ball scars is significantly high as compared to the virgin film surfaces (Fig. 6a). Furthermore, this ratio is 2.71 and more prominent in vacuum tribology condition as compared to 1.95 in ambient condition. This fact clearly indicates more transformation of sp^3^ into a-C and/or sp^2^ phase and formation of tribolayer at the ball counterbodies during sliding process in vacuum conditions. However, the nature of the Al2p peak is similar in both tribo-conditions as shown in Fig. 12e.

It is worth mentioning that the above chemical and phase composition analysis using EDX and XPS is a model experimental performance at the ball scar of Al_2_O_3_ while sliding against the UNCD(0%H_2_) films in both ambient and vacuum conditions. Moreover, Raman spectra of ball scar and wear track formed in UNCD(6%H_2_) films in ambient and vacuum conditions are shown in Figs 8 and 9 respectively. The results clearly showed the chemical stability of UNCD films in ambient tribo-condition without much formation of carbonaceous (a-C/graphitized) transferlayer at the ball scar (Fig. 8). More transferlayer is observed at the Al_2_O_3_ ball scar in vacuum condition as indicated by enhancement in the Raman intensity of D and G bands. This also affects the chemical stability of wear track as shown in Fig. 9. These above evidences are sufficient enough to analyze the phase composition in tribo-contact interfaces.

The deformation of sliding bodies is associated with the chemical transformations at the sliding interfaces. In this aspect, the deformation of wear tracks in UNCD(6%H_2_) and UNCD(0%H_2_) films was less in the ambient as compared to vacuum tribology conditions (Figs 5SI and 6SI). FESEM image of the virgin film surfaces is also shown in Fig. 7SI. Though, the feature of nanostructured diamond is clearly preserved in both the wear tracks but it is more marked in the case of UNCD(6%H_2_) films (Fig. 5SIa). However, transferlayer was formed locally in both the wear tracks and the locations are shown by the arrows in high-resolution image, indicating layers of deformed Al_2_O_3_ particles. This is more evident at the edge of the wear tracks where cracks within thick layer were observed. More deformed wear track with severe plowing wear of UNCD(6%H_2_) films was observed in high vacuum tribology condition (Fig. 6SIa). The wear track is plastically deformed and feature of nanostructured diamond disappeared as shown in the high-resolution FESEM image. However, abrasive wear mechanism is governed in UNCD(0%H_2_) films at similar tribology conditions (Fig. 6SIb). In both the films, the Ti-alloy surfaces are not exposed by deformation, in both tribology conditions and the extent of deformation is well within the film surfaces. This showed the extremely high wear resistance of these films in both ambient and high vacuum conditions even after 5 × 10^4^ cycles. Thus, deep wear marks were not observed in inset image of Fig. 7.

### Tribology mechanism

The above results showed that the high contact stress is not responsible for the conversion of UNCD structure into a-C and graphite phases. In the beginning of the test, contact stress was comparatively high 35 MPa but the structural conversion was not noticed in Raman spectra (Fig. 3SIa_i_). Contact stress continuously reduced with sliding cycles due to the increase in contact area which was related to the deformation of the ball (Fig. 8SI). This indicates that high stress is not the operative mechanism for structural conversion of UNCD into a-C and graphite phases. Such conversion was noticed earlier by Bouchet *et al*. in NCD film even at mild tribo-contact condition^16^. However, we have presented here mechanistic analysis of contact stress and conversion of UNCD to sp^2^/a-C phases. Reasonably, the possible mechanism for such a conversion is described by sliding induced fatigue^40^ in UNCD films where the structural conversion takes place by micro-cracking. Other possible reasons for such conversion could be related to the flash temperature^41^ generated due to the kinetic energy of the sliding bodies.

On the basis of above results, friction and wear mechanism in UNCD films in two different atmospheres could be possibly explained by elemental composition, phase composition and transferlayer characteristic analysis at the contact interfaces. XPS results showed that in UNCD(6%H_2_) films, the sp^2^ phase fraction is negligible at the film surfaces i.e., 1.8 at%. However, this value increased to 14.4 at% in UNCD(0%H_2_) films. In contrast, the sp^3^ fraction is almost similar in both films (Table 1SI). However, sp^2^ phase analyzed by AES is much higher on top of the surface in both these films which do not have much influence on the friction as mentioned above. The contaminated top surface was removed easily during run-in regime and it is not considered for tribological analysis. Therefore, sp^2^ and sp^3^ fraction calculated by XPS is relevant for friction and wear analysis. In ambient atmosphere, both these films showed almost similar trend of friction coefficients. This could be explained by the influence of ambient chemical species at tribo-contact areas^19–21^. Here, water molecules and vapor from moisture passivate the unoccupied carbon bonds of sp^3^ and sp^2^ phases at the tribo-contact regions, forming carbon-oxygen complexes. The mixture of oxidized sp^3^ with a-C and sp^2^ phase present at the contact as a tribolayer may react mainly with the oxygen from the moisture. It could be important to note that in ambient tribo-conditions, the formation of carbon transferlayer was not significant but oxygen was fully developed at the ball scar as evident from EDX (Fig. 10g and h) and XPS (Fig. 12a) analysis. Also, micro-Raman results of wear track at various locations of UNCD(6%H_2_) and UNCD(0%H_2_) films clearly showed stable chemical feature of UNCD structure which is almost similar to the virgin film surfaces (Fig. 8a_i_ and b_i_). However, a very weak signal of a-C layer was observed at ball scar in UNCD (6% H_2_)/Al_2_O_3_ sliding combinations (Fig. 8a_ii_). Furthermore, this layer at the ball scar was very less in UNCD(0%H_2_)/Al_2_O_3_ sliding combinations as shown by micro-Raman (Fig. 8b_ii_) and EDX analysis (Fig. 10). The XPS survey spectra support the same feature which showed comparatively less carbon counts (Fig. 12a_i_). Generally, such stability of the UNCD structure in the tribo-stressed condition is derived by passivation effect in ambient and moist conditions^19–22^. In stressful sliding, plastic deformation may take place and some of the sp^3^ and sp^2^ phases may collapse due to fatigue. However, these phases are instantaneously passivated by the carbon-hydrogen and carbon-oxygen complexes. This further restricts the transformation of UNCD to a-C and graphite phases. Therefore, low friction and wear in ambient condition is described by the passivation effect where two sliding planes could have minimum shear resistance. In contrast, the a-C transferlayer was fully developed on ball scar (Fig. 9a_ii_ and b_ii_) and oxygen at% also decreased at the ball scar region in vacuum conditions (Figs 11 and 12). In this case, C-C strong sliding planes could be expected at the contact interfaces, enhancing the frictional resistance. This fact points out that UNCD films behave differently in high vacuum tribology condition where external passivation mechanism does not work and therefore, in this condition, intrinsic properties of material dominate to influence the tribological behavior. The summary of the above mentioned mechanism is illustrated in Fig. 9SI.

The friction coefficient in UNCD(6%H_2_) films is very less, up to 1 × 10^4^ sliding cycles, in vacuum condition. After that, this value gradually increased to higher magnitude. The SIMS analysis showed a large amount of hydrogen in these films (Fig. 2 and 1SI). Here, low friction region is derived by the intrinsically present hydrogen in the films which provides passivation of the carbon dangling bonds in the vacuum condition. This fact is well described in hydrogenated DLC and hydrogenated diamond nanostructured materials^42,43^. The UNCD(6%H_2_) films showed improved antifriction behavior as these contain small fraction of a-C/sp^2^ phase as evident from XPS analysis. Therefore, the intrinsically present hydrogen in the films is the effective reason for low friction mechanism in vacuum conditions which sustains the stability and activates the passivation mechanism. In the beginning of the sliding cycles (2 × 10^3^), no change in the chemical structure of UNCD(6%H_2_) films in the wear track was observed and carbon signal at the ball scar was negligible (Figs 3SIa_i_ and 3SIb_i_). However, a-C layer was gradually formed at the ball scar with increasing sliding cycles (Figs 3SIa_ii_ and 3SIb_ii_). Furthermore, at 1 × 10^4^ cycles, the a-C signal (a broader peak of D and G band) evolved at the ball scar. Also, the other peaks relevant to UNCD structure were not observed in this case (Figs 3SIa_iii_ and 3SIb_iii_). At low friction regime, the spectral changes showing a-C were localized at few locations of the ball scar which are densely distributed at the end of the sliding cycles of 5 × 10^4^ (Fig. 9a_ii_). This result clearly indicates the chemical affinity of a-C/graphite phase towards the Al_2_O_3_ ball. Therefore, it was perceived that the conversion of UNCD to a-C/graphite phase was observed at high friction region. This fact was well studied in DLC sliding systems which showed the formation of covalently bonded shear plane due to the transformation of saturated hydrogenated DLC into unsaturated sp- and sp^2^- phases^9^. Thus, in DLC films, it was shown that the amorphization and graphitization do not assist in reducing the friction in high vacuum condition^42,44^. The reason for the gradual formation of a-C and increase in friction value is possibly explained by desorption of hydrogen in the continuous tribo-stressed condition in high vacuum. The similar mechanism in high vacuum tribo-condition was explained by Gao *et al*. in hydrogenated DLC film using mass spectrometer which showed the desorption of hydrogen at longer sliding cycles^7^. The intrinsic passivation mechanism in the vacuum conditions stops to work after desorption of hydrogen. This causes to increase the frictional resistance and leads to the transformation of UNCD structure into a-C/graphite phases. In contrast, stable high friction value is observed throughout the whole sliding cycles in UNCD(0%H_2_) films in vacuum conditions. Here, carbon transferlayer was significantly formed at the ball scar as evident from micro-Raman spectroscopy (Fig. 9b_ii_), EDX (Fig. 11) and XPS (Fig. 12) analyses. This may induce strong adhesive interaction between the sliding surfaces and thus resulted in high friction values. It is important to note that intrinsic hydrogen was limited in these films to passivate the sliding interfaces in vacuum conditions.

On the other hand, the amount of hydrogen in UNCD(0%H_2_) films is less and therefore, passivation mechanism of carbon dangling bonds in the tribo-contact region is limited in the beginning of the sliding cycles. Therefore, the friction value is higher during the initial sliding cycles and does not change further with time. The antifriction mechanism does not operate through graphitization even though these films contain high fraction of sp^2^ phase (14.4 at%). It is well known that the antifriction and antiwear mechanism of graphite takes place in ambient conditions where water vapor get adsorbed in the tribo-contact region and reciprocates to reduce the shear resistance of graphite sheets^45,46^. This basically describes the reduction of shear energy in the contact region due to basal plane passivation. However, in high vacuum and inert conditions, shear resistance of the graphite plane increases due to the lack of passivation which results in high friction and wear. Further, low shear resistance of graphite planes are restricted in UNCD(0%H_2_) films owing to less amount of hydrogen content. High friction significantly transformed the UNCD structure into a-C and graphite phase in the wear track resulting the appearance of prominent a-C structure at the ball scar. From these results, it was noticed that ambient conditions preserved the UNCD structure under tribo-stressed conditions and in turn, tribological properties were enhanced. However, high vacuum tribo-conditions transformed the UNCD structure into a-C/graphite phases forming transferlayer of the same phase on ball scar. In this condition, antifriction and antiwear properties were lost due to the interaction of dangling bonds which forms stronger bonding between the sliding planes^9^.

The trend of friction coefficient in run-in regime in high vacuum and ambient conditions is similar in both the films. However, in both tests conditions, the magnitude of friction in run-in regime is less in high vacuum. Therefore, this could not be explained only by the residual roughness of the film surface. In high vacuum conditions, the physisorbed contamination such as the moisture and water molecules desorbs at the surface resulting in reduced friction coefficient.

## Conclusions

Technologically high-quality and novel UNCD films with desired microstructure and chemical properties were deposited on Ti-alloy using MPECVD technique. The tribological properties of these two distinct UNCD films, with respect to microstructure and chemical properties were analyzed in ambient and high vacuum conditions for understanding their sustainable tribological feature. Friction coefficient of UNCD films containing excess hydrogen was less (0.1) in high vacuum tribology condition up to the limited sliding cycles. This was explained by the passivation of dangling carbon bonds at the sliding interfaces by the hydrogen already present in the film. However, UNCD films with less hydrogen concentration showed high friction value in high vacuum condition due to limited intrinsic passivation mechanism of carbon dangling bonds. This resulted in the formation of a-C transferlayer at the ball scar as evident from comprehensive characterization using Raman spectroscopy, EDX and XPS analysis. This ultimately leads to the increase in frictional resistance due to the formation of stronger bonds across the sliding interfaces of a-C tribofilm. Thus, it was confirmed that the disordered graphite and a-C phase formation at sliding interfaces was responsible for the increase in friction coefficient in vacuum conditions. However, both these films showed less friction values with similar trend in ambient tribology conditions which was explained by the presence of natural passivation mechanism by atmospheric chemical species. In this condition, the formation of a-C tribofilm was limited.

## Experimental Methods

### Film deposition

The UNCD films were grown on mirror polished Ti-alloy substrates in microwave plasma enhanced chemical vapor deposition (MPECVD) system (2.45 GHz 600 IPLAS-CYRANNUS). Two different UNCD films were deposited in H_2_(6%)/CH_4_(2%)/Ar(rest) or H_2_(0%)/CH_4_(2%)/Ar(rest) plasma at a pressure of 150 Torr and 130 Torr, respectively. The microwave power of 1200 W and a substrate temperature of 550 °C were used during the deposition processes. For simplicity, the films deposited in H_2_(6%)/CH_4_(2%)/Ar and H_2_(0%)/CH_4_(2%)/Ar plasma were designated as UNCD(6%H_2_) and UNCD(0%H_2_) films, respectively.

### Characterization techniques

The surface topography of these films were analyzed by AFM (Park XE-100). The surface morphology and wear tracks were examined by FESEM (Zeiss Supra 55). Depths profiling elemental analysis of C, H and Ti in both films were carried out by time of flight SIMS (TOF-SIMS5, ION-TOF GmbH) using Bismuth (Bi^+^) ion source. Two-dimensional (2D) and three-dimensional (3D) image of depth profiling were generated for quantitative elemental analysis. Detailed microstructure and local bonding of both the films were analyzed by HRTEM (JEOL 2100 F) and EELS (Getan, Enfina). For these analysis, bulk portion of the film was used to avoid the surface contamination. The TEM and EELS samples were prepared by mechanical grinding (or dimpling) followed by Ar-ion milling of the original films. In this process, the final thin foil for the TEM analysis, mainly contains the materials in the region near the surface of the UNCD films. EELS spectra were taken in diffraction mode from circular sample areas approximately 0.4 mm in diameter as defined by the selected area aperture, a camera length of 84 mm and a 2 mm spectrometer entrance aperture providing a collection semi-angle (*β*) of 3.4 mrad and a convergence angle (*α*) of 21.3 mrad. The surface chemical structure of the films was investigated by XPS and Auger spectroscopy (ESCALAB 250) using monochromatic AlKα radiation. The chemical structure of films, wear tracks and ball scars were examined by the micro-Raman spectrometer (Andor SR-500i-C-R, λ = 532 nm). The surface chemical analysis and elemental analysis on ball scars were carried out using XPS and EDX-imaging, respectively. Usually, backscattered electron images in the SEM display compositional contrast resulting from different atomic number elements and their distribution on the sample surface. Energy dispersive X-ray (EDX) spectroscopy allows one to identify the particular elements present on the surface and their relative proportions. The EDX signal is obtained in pixel which is defined in colors for each element, and then it is superimposed to get the total information in a defined scanned area. Basically pixel size defines the quantity of elements which is then converted into the percentage for easy understanding using software. Moreover, pixel is associated to X-ray signal received by the EDX detector.

### Tribology test

The friction and wear behaviors of these films were measured in high-vacuum tribometer (Anton Paar, Switzerland) operating in a circular rotation mode of pin-on-disc configuration. The tribology tests were performed along the circular path of diameter 2 mm and up to a sliding cycles of 5 × 10^4^. An Al_2_O_3_ ball with 6 mm diameter and average roughness ~45 nm was used as a sliding counterbody. The tests were carried out in both ambient and high vacuum (3 × 10^−6^ mbar) tribology conditions.

SIMS 2D and 3D depth profiling images of UNCD films; XPS survey spectra of UNCD films; Raman spectra at various locations of wear track and ball scars; Raman spectra of Al_2_O_3_ ball; Wear track morphology formed in ambient condition; Wear track morphology formed in high vacuum condition; Surface morphology of films; Contact stress with sliding cycles; Summary of transferlayer formation. This material is available free of charge via the Internet at http://www.nature.com/srep.

## Electronic supplementary material

Supplementary Information
