# Supplementary material for: Tribological Properties of Ultrananocrystalline Diamond Films: Mechanochemical Transformation of Sliding Interfaces
Source: Sci Rep. 2018 Jan 10;8:283. doi: 10.1038/s41598-017-18425-4 (PMC5762651; doi:10.1038/s41598-017-18425-4)
Supplement: Supplementary file 1 — Supplementary Information [file 41598_2017_18425_MOESM1_ESM.doc]

Supporting Information

**Tribological properties of ultrananocrystalline diamond films: Mechanochemical transformation of sliding interfaces**

Revati Rani^1^, Kalpataru Panda^2^, Niranjan Kumar^1*^, Kozakov Alexey Titovich^3^, Kolesnikov Vladimir Ivanovich^4^, Sidashov Andrey Vyacheslavovich^4^ & I-Nan Lin^5^

^1^Indira Gandhi Centre for Atomic Research, HBNI Kalpakkam 603102, Tamil Nadu, India.

^2^Center for Nanomaterials and Chemical Reactions, Institute for Basic Science (IBS), Daejeon

34141, Korea.

^3^Southern Federal University, Rostov on Don, 344006, Russian Federation.

^4^Rostov State Transport University, Rostov-on-Don, 344008, Russian Federation.

^5^Department of Physics, Tamkang University, New-Taipei 251, Taiwan, Republic of China.

**Figure contents:**

**Figure 1-SI.** 2D and 3D depth profiling image of H^+^, C^+^ and Ti^+^ in UNCD(6%H_2_) film and UNCD(0%H_2_) film.

**Figure 2-SI.** Survey XPS of (a) UNCD(6%H_2_) film and (b) UNCD(0%H_2_) film.

**Figure 3-SI.** Raman spectra at various locations: of (a_i_, a_ii_, a_iii_) wear track and (b_i_, b_i_, b_iii_) ball scar in the sliding combination of UNCD(6%H_2_)/Al_2_O_3_ at sliding cycles of (a_i_, b_i_) 2×10^3^, (a_ii_, b_ii_) 5×10^3^, and (a_iii_, b_iii_) 1×10^4^, tribology parameters: High vacuum (3×10^–6^ mbar), Load: 1 N, Sliding speed: 100 rpm (linear speed: 1.04 cm/s), Ball: Al_2_O_3_ (6 mm dia.).

**Figure 4-SI.** Raman spectra of the virgin Al_2_O_3_ ball surface.

**Figure 5-SI.** Wear track morphology of (a) UNCD(6%H_2_) film and (b) UNCD(0%H_2_) film; tribology parameters: Ambient, Load: 1 N, Sliding speed: 100 rpm (linear speed: 1.04 cm/s), Ball: Al_2_O_3_ (6 mm dia.).

**Figure 6-SI.** Wear track morphology of (a) UNCD(6%H_2_) film and (b) UNCD(0%H_2_) film; tribology parameters: High vacuum (3×10^–6^ mbar), Load: 1 N, Sliding speed: 100 rpm (linear speed: 1.04 cm/s), Ball: Al_2_O_3_ (6 mm dia.).

**Figure 7-SI.** Surface morphology of (a) UNCD(6%H_2_) film and (b) UNCD(0%H_2_) film.

**Figure 8-SI.** Relationship of contact stress with sliding cycles, considering deformation of the ball

**Figure 9-SI.** Chemical analysis of transferlayer and friction trend in ambient and high vacuum condition of UNCD(0%H_2_) film

**Table contents:**

**Table 1-SI.** Proportion of sp^3^ and sp^2^ with other chemical complexes in UNCD film surface defined by high resolution C1s spectra.

**Table 2-SI.** Chemical shift of oxygen complexes in high resolution O1s spectra of the UNCD film surface.


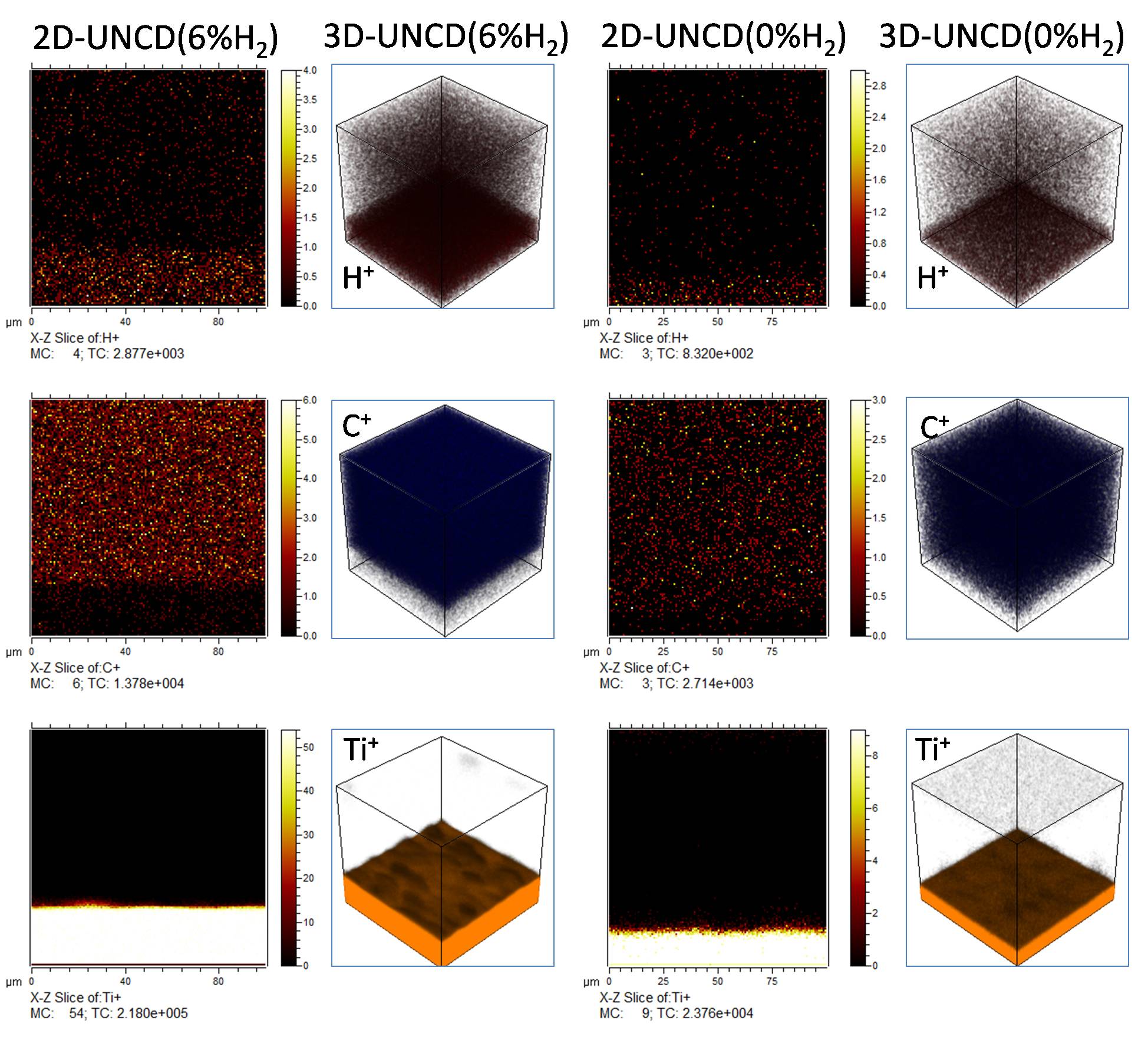


**Figure 1-SI.** 2D and 3D depth profiling image of H^+^, C^+^ and Ti^+^ in UNCD(6%H_2_) film and UNCD(0%H_2_) film.


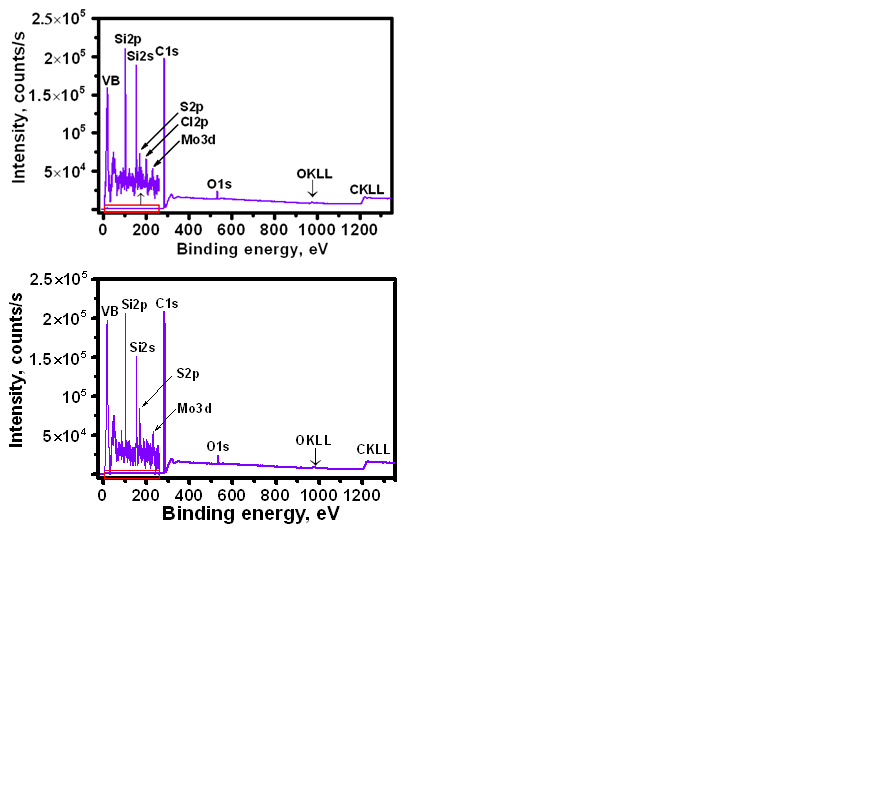


**(a)**

**(b)**

**Figure 2-SI.** Survey XPS of (a) UNCD(6%H_2_) film and (b) UNCD(0%H_2_) film.

**Table 1-SI.** Proportion of sp^3^ and sp^2^ with other chemical complexes in UNCD films surface defined by high resolution C1s spectra

| Chemical component | Chemical network | Quantity of hydrogen in plasma (%) | | | |
| --- | --- | --- | --- | --- | --- |
|  |  | UNCD(6%H_2_) | | UNCD(0%H_2_) | |
|  |  | Binding energy, eV | Atomic fraction, % | Binding energy, eV | Atomic fraction, % |
| A | C-C (sp^2^) | 284.5 | 1.8 | 284.4 | 14.4 |
| B | С – С (sp^3^) | 285.0 | 71.2 | 284.9 | 71.1 |
| C | C-COO  CH_3_COH | 285.7 | 21.3 | 285.7 | 14.4 |
| D | CH_2_ – O | 286.5 | 4.7 | 286.7 | 1.4 |
| E | CH_2_ - O | 287.9 | 1.4 | ---- | ---- |

**Table 2-SI.** Chemical shift of oxygen complexes in high resolution O1s spectra of the UNCD films surface

| Chemical component | Chemical network | Quantity of hydrogen in plasma (%) | | | |
| --- | --- | --- | --- | --- | --- |
|  |  | UNCD(6%H_2_) | | UNCD(0%H_2_) | |
|  |  | Binding energy, eV | Atomic fraction, % | Binding energy, eV | Atomic fraction, % |
| A | C-COO  CH_3_COH | 530.6 | 2.1 |  |  |
| А/ | Weakly bonded oxygen atom in the form of superoxide, peroxide and ozonide | ---- | ---- | 531.6 | 8.2 |
| B | OH- groups | 532.6 | 56.3 | 532.4 | 74.9 |
| C | OH- groups,  H_2_O | 533.8 | 31.4 | 533.9 | 16.9 |
| D | OH- groups,  H_2_O | 534.4 | 10.2 | ---- | ---- |


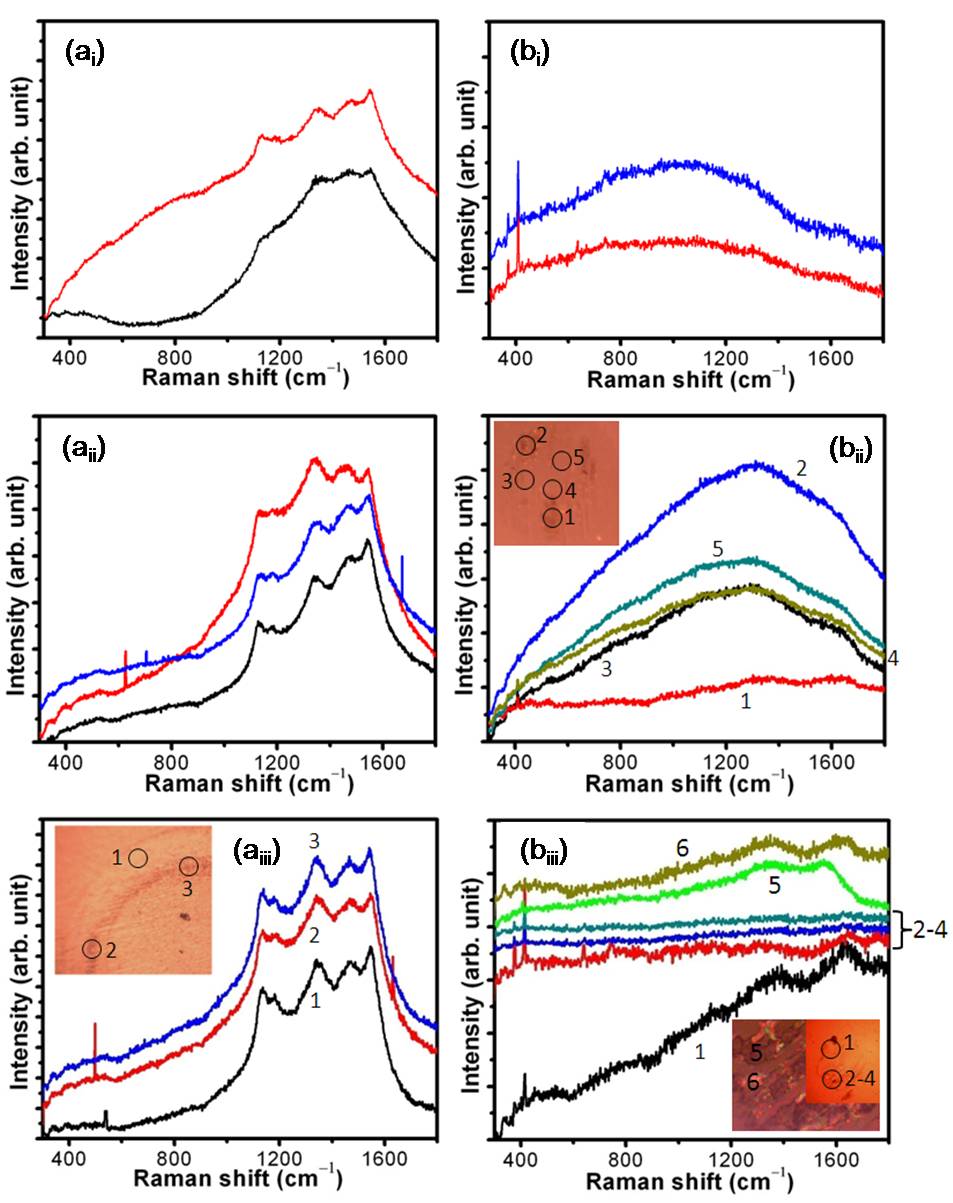


**Figure 3-SI.** Raman spectra at various locations: of (a_i_, a_ii_, a_iii_) wear track and (b_i_, b_i_, b_iii_) ball scar in the sliding combination of UNCD(6%H_2_)/Al_2_O_3_ at sliding cycles of (a_i_, b_i_) 2×10^3^, (a_ii_, b_ii_) 5×10^3^, and (a_iii_, b_iii_) 1×10^4^; tribology parameters: High vacuum (3×10^–6^ mbar), Load: 1 N, Sliding speed: 100 rpm (linear speed: 1.04 cm/s), Ball: Al_2_O_3_ (6 mm dia.).


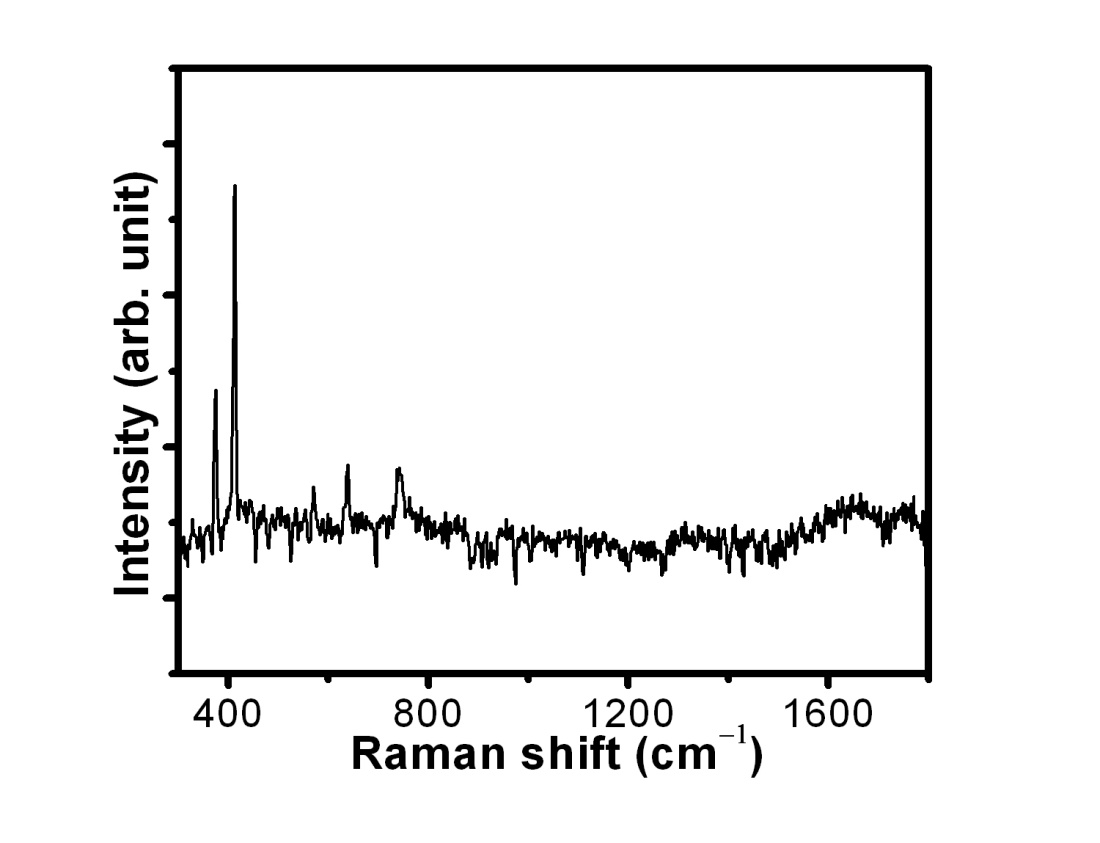


**Figure 4-SI.** Raman spectra of the virgin Al_2_O_3_ ball surface.


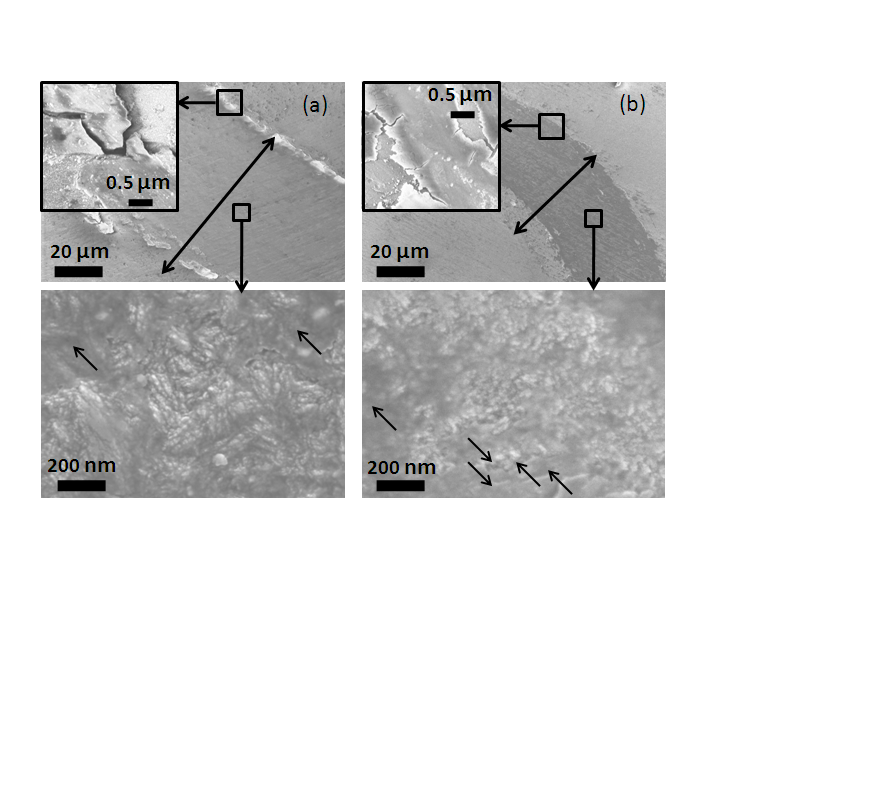


**Figure 5-SI.** Wear track morphology of (a) UNCD(6%H_2_) film and (b) UNCD(0%H_2_) film; tribology parameters: Ambient, Load: 1 N, Sliding speed: 100 rpm (linear speed: 1.04 cm/s), Ball: Al_2_O_3_ (6 mm dia.).


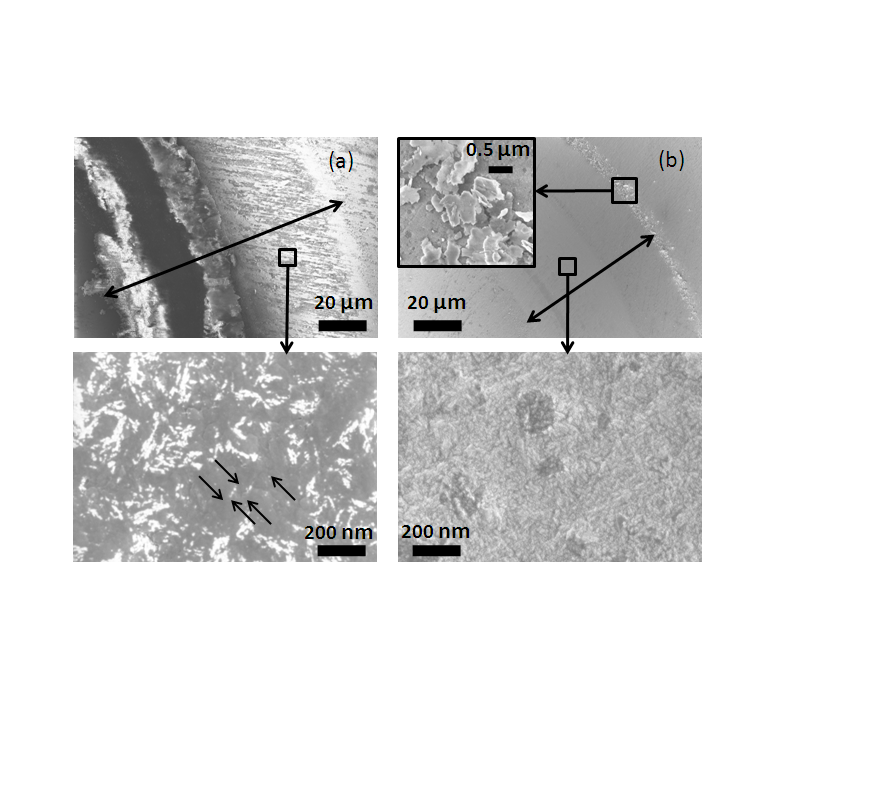


**Figure 6-SI.** Wear track morphology of (a) UNCD(6%H_2_) film and (b) UNCD(0%H_2_) film; tribology parameters: High vacuum (3×10^–6^ mbar), Load: 1 N, Sliding speed: 100 rpm (linear speed: 1.04 cm/s), Ball: Al_2_O_3_ (6 mm dia.).


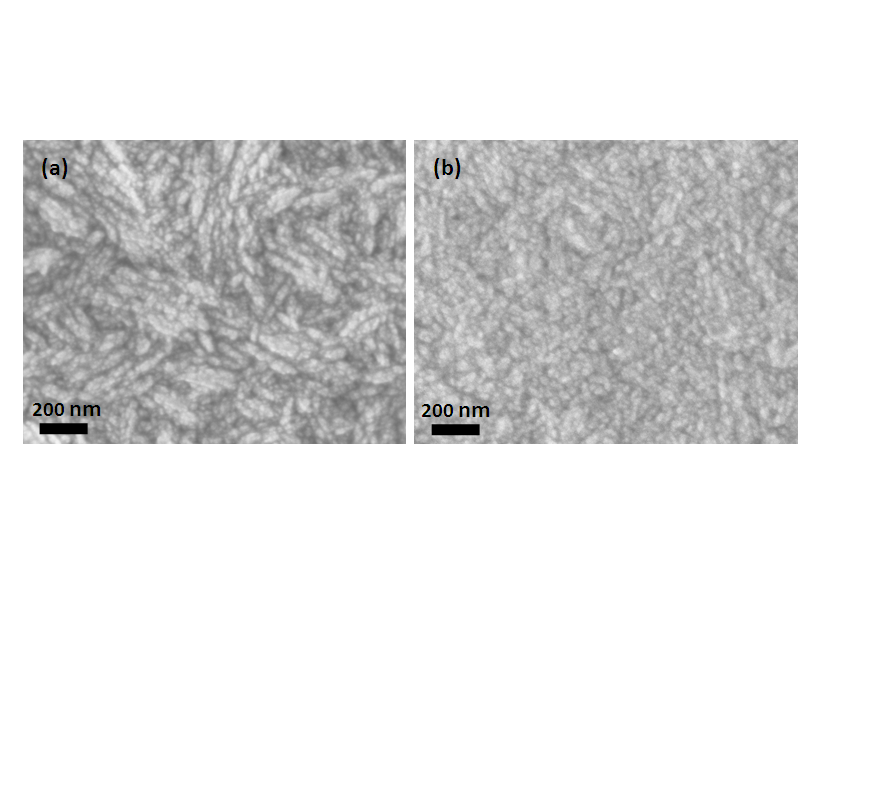


**Figure 7-SI.** Surface morphology of (a) UNCD(6%H_2_) film and (b) UNCD(0%H_2_) film.


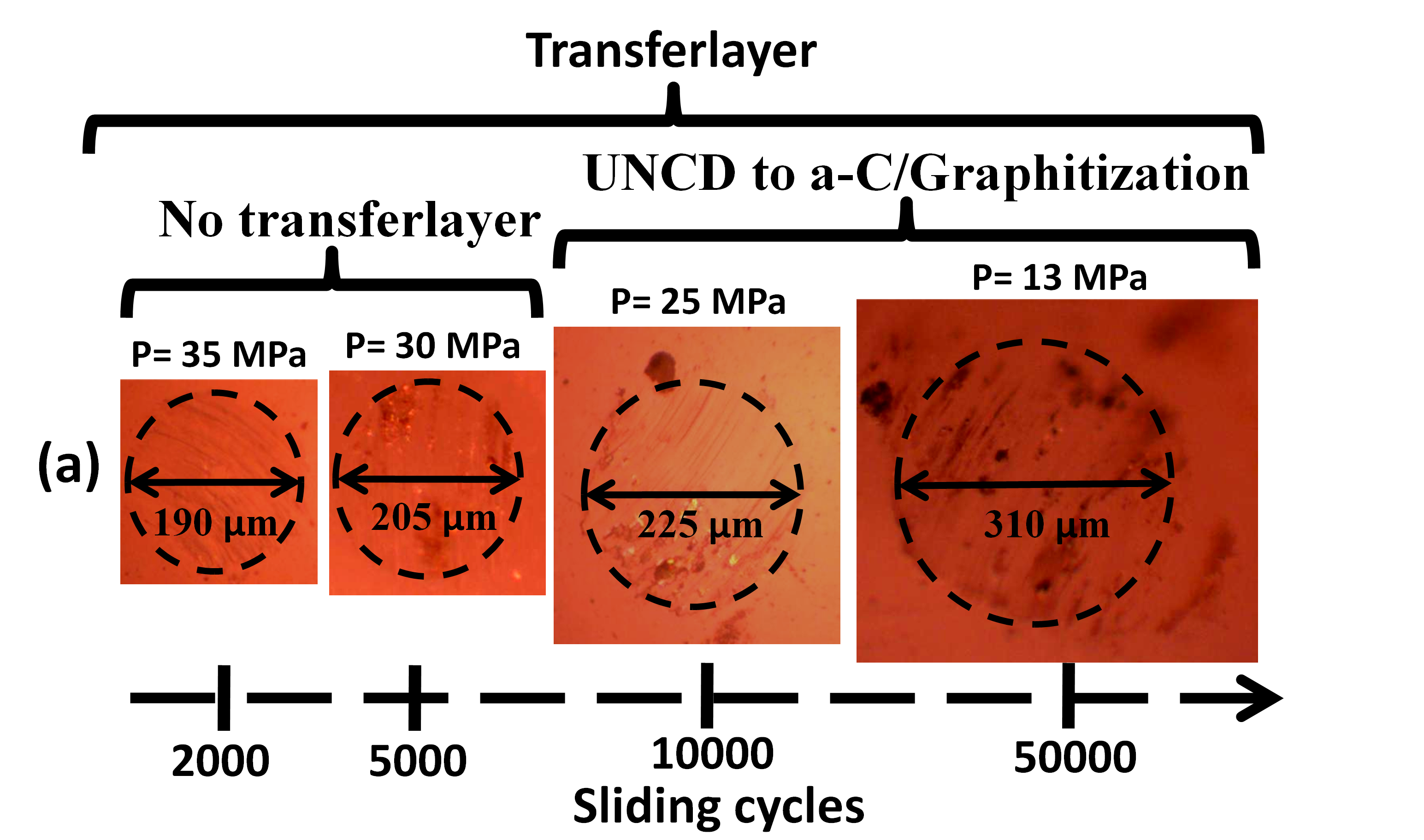


**Figure 8-SI.** Relationship of contact stress with sliding cycles, considering deformation

of the ball


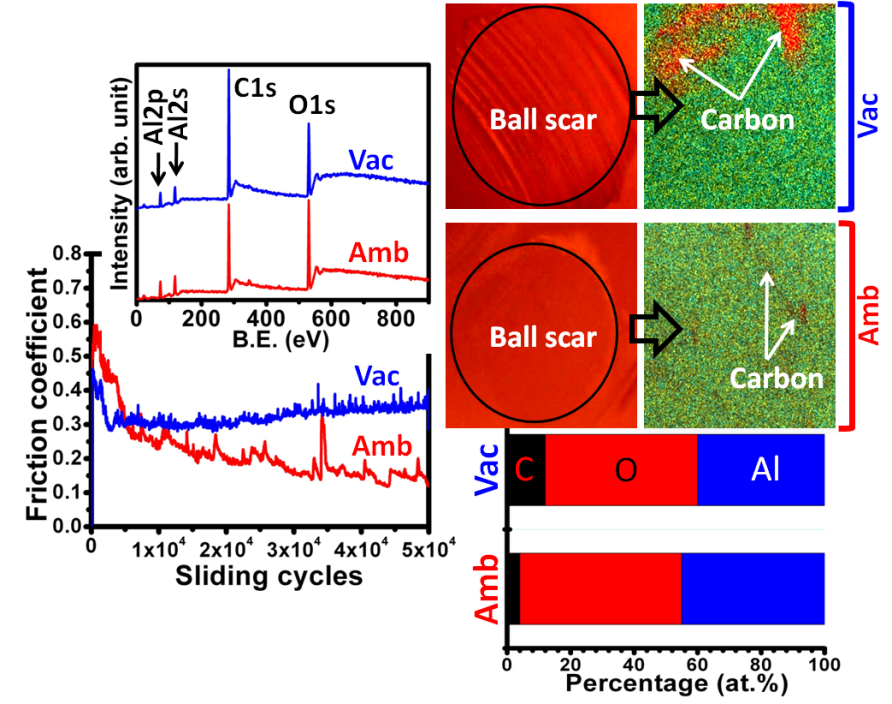


**Figure 9-SI.** Chemical analysis of transferlayer and friction trend in ambient and high vacuum condition of UNCD(0%H_2_) film
